# Identifying shared polygenic risk across cancers

**DOI:** 10.1371/journal.pgen.1012308

**Published:** 2026-09-18

**Authors:** Jiaqi Hu, Maiyier Muheyati, Leqi Xu, Andrew DeWan, Hongyu Zhao

**Affiliations:** 1 Department of Chronic Disease Epidemiology, Yale School of Public Health, New Haven, Connecticut United States of America; 2 Department of Biostatistics, Yale School of Public Health, New Haven, Connecticut, United States of America; 3 Program of Computational Biology and Bioinformatics, Yale University, New Haven, Connecticut United States of America; Newcastle University, UNITED KINGDOM OF GREAT BRITAIN AND NORTHERN IRELAND

## Abstract

**Background:**

Shared genetic susceptibility across cancers has been reported but is generally modest at the genome-wide level. Whether such shared polygenic risk exhibits structured convergence at regional or functional levels remains unclear. We investigated shared genetic risk across cancers by integrating local genetic correlation analyses with cross-cancer polygenic risk score (PRS) associations.

**Methods:**

We estimated pairwise local genetic correlations across 16 specific cancers and one pan-cancer phenotype using SUPERGNOVA. Genome regions harboring multiple cancers with mutually correlated local genetic effects were annotated using genetic correlations with non-cancer phenotypes and associations from the GWAS Catalog. In parallel, cross-cancer PRS associations were evaluated, and significant cancer pairs were identified. Genome-wide PRSs for selected pairs were further decomposed into pleiotropy-informed and pathway-specific components to assess functional enrichment of shared polygenic risk.

**Results:**

Genome-wide genetic correlation analyses identified 20 significantly correlated cancer pairs, whereas local analyses revealed 82 regions with shared genetic signals across 66 cancer pairs. Five regions exhibited mutually correlated cancer clusters, with enrichment in functional domains such as inflammatory functions. Cross-cancer PRS analyses identified five cancer pairs with shared polygenic risk. Decomposition of PRSs indicated that these cross-cancer associations were enriched in specific pleiotropy groups and immune-related pathways rather than reflecting diffuse genome-wide overlap.

**Conclusion:**

Our findings demonstrate that although shared genetic susceptibility across cancers is limited at the genome-wide level, it becomes evident when examined at regional and polygenic scales. Integrating local genetic correlation and PRS decomposition analyses reveals structured patterns of shared genetic risk, providing a framework for investigating cross-cancer polygenic susceptibility.

## Author summary

Many cancers are known to share inherited genetic risk, but it remains unclear where this genetic overlap occurs and what biological mechanisms underlie it. Identifying these shared genetic influences may reveal common processes involved in cancer development and could ultimately improve cancer risk prediction and prevention. In this study, we analyzed genetic data from 24 specific cancer types and a pan-cancer phenotype to investigate shared inherited cancer risk. In addition to assessing genetic overlap across the genome, we examined specific genomic regions and groups of genes involved in related biological functions. We found that most cancers showed only limited shared genetic risk at the genome-wide level. However, many pairs of cancers shared genetic influences within particular genomic regions and biological pathways. These findings suggest that different cancers may arise through overlapping genetic mechanisms, even when their overall genetic similarity is low. Overall, this study provides a framework for identifying shared genetic susceptibility across cancer types and offers new insight into biological processes that contribute to cancer development, with potential implications for future cancer prevention and risk prediction.

## Introduction

Genetic factors contribute substantially to cancer susceptibility. Evidence from a large Nordic twin cohort demonstrates that approximately one-third of the variance in overall cancer risk is attributable to heritable factors (heritability [h^2^] = 33%, 95% CI: 30%-37%) [1]. Genome-wide association studies (GWASs) have identified numerous common variants associated with risk across diverse cancer types [2–4]. Notably, some variants exhibit pleiotropy, i.e., they are independently associated with two or more cancers. For example, rs78378222 in *TP53* has been implicated in the risk of breast cancer, skin cancer, and several other malignancies [2,5]. These observations suggest shared genetic architecture across cancers and highlight the importance of systematically characterizing cross-cancer genetic overlap.

Efforts to quantify shared genetic susceptibility across cancers—typically measured through genetic correlations— have consistently shown that genome-wide overlap is generally modest. Sampson et al. (2015) evaluated pairwise genetic correlations among 13 cancers and identified significant correlations for only a few pairs, such as bladder and lung cancers, indicating limited shared heritable risk at the genome-wide level [6]. Subsequent studies leveraging larger GWAS datasets have reported similarly low genetic correlations [7,8]. Nevertheless, some cancer pairs exhibit local signals despite weak or nonsignificant genome-wide correlations. For instance, shared risk at the chromosome 9p21 region has been observed across several cancers [7], suggesting that genetic overlap may be concentrated within specific genomic regions rather than distributed broadly across the genome. Furthermore, polygenic risk scores (PRSs), which aggregate effects of risk variants, have revealed cross-cancer associations that are not captured by the global genetic correlation estimate [9], pointing to additional cross-cancer relationships.

Although prior studies have quantified the generally modest genetic overlap across cancers and highlighted that shared signals may be confined to specific genomic regions, the underlying biological mechanisms of this overlap remain insufficiently understood. In particular, it is unclear whether the variants or loci shared across cancers converge on common oncogenic pathways or reflect distinct, context-dependent mechanisms. Moreover, most existing analyses have examined cancer pairs in isolation, limiting the ability to characterize broader patterns of shared genetic architecture. As a result, a comprehensive, network-level understanding of how multiple cancers are genetically interconnected has yet to be fully established.

In this study, we aimed to characterize shared genetic susceptibility across cancers by integrating regional correlation analyses with polygenic risk evaluation. We decomposed the genome-wide sharing into local signals through local genetic correlations and PRS decompositions. Our results confirmed that the shared genetic architecture across cancers on the genome-wide scale is modest, but the overlapped genetic risk is enriched in certain functions, such as immune-metabolic pathways.

## Methods

### Overview of methods

Using GWAS summary statistics for 24 specific cancers and one pan-cancer phenotype, we first characterized shared genetic susceptibility across cancers by estimating genetic correlations at both genome-wide and regional scales. To provide functional context for regional genetic sharing, we further annotated correlated regions by evaluating their genetic correlations with non-cancer phenotypes and by linking variants within these regions to previously reported associations in the GWAS Catalog.

We next examined pairwise polygenic overlap using PRSs in UK Biobank (UKB) participants. Genome-wide PRSs were used to identify cancer pairs exhibiting significant cross-cancer associations. To further investigate the potential sources of shared polygenic risk, PRSs were decomposed into components with different functions, enabling attribution of cross-cancer associations to pleiotropy groups and biological pathways.

### GWAS summary statistics

We downloaded GWAS summary statistics for 24 specific cancers and one pan-cancer phenotype, restricting analyses to studies of European ancestry and excluding datasets that included UKB participants [2–4,10,11]. Sample sizes and study details for all cancer phenotypes are provided in S1 Table. Briefly, the breast cancer GWAS included 133,384 cases and 113,789 controls [2], the prostate cancer GWAS included 79,194 cases and 61,112 controls [3], the lung cancer GWAS comprised 44,069 cases and 68,712 controls [4], and the ovarian cancer GWAS consisted of 25,509 cases and 40,941 controls [10]. The remaining 21 cancer GWASs were derived from the FinnGen R12 release [11], which used a shared set of cancer-free controls (except for sex-specific cancers including cervix uteri and testis cancers) and included between 152 cases for nasopharyngeal cancer and 121,495 cases for the pan-cancer phenotype. The pan-cancer was defined as diagnosis of any malignant neoplasm. FinnGen is a large-scale national research initiative integrating genotype data from over 500,000 Finnish biobank participants with comprehensive longitudinal health registry data to study disease susceptibility.

All summary statistics underwent quality control procedures. We excluded non-autosomal variants, insertion-deletion polymorphisms, and variants not present in the UKB genotype. For multi-allelic variants, a single allele was retained based on the smallest association p-value. The cleaned summary statistics were then harmonized and processed using LD score regression (LDSC) [12] to estimate SNP-based heritability, restricting analyses to variants with minor allele frequency (MAF) ≥ 0.001. Cancers with heritability not significantly larger than 0 were excluded from downstream analyses.

### UK biobank data

Individual-level data from the UKB were used for PRS analyses. UKB is a large prospective cohort study that has enrolled over 500,000 participants aged 40–69 years across the United Kingdom [13]. Cancer case status was ascertained using a combination of self-reported diagnoses and linked hospital records, incorporating International Classification of Diseases, Ninth and Tenth Revisions (ICD-9 and ICD-10), as well as Office for National Statistics Classification of Interventions and Procedures (OPCS) codes. The specific codes used to define cancer outcomes are provided in S2 Table. Individuals with any cancer diagnosis were classified as cases, and controls were defined as participants without a recorded diagnosis of any cancers.

Analyses were restricted to unrelated participants of genetically inferred European ancestry [14]. We used phase III UKB genotype data, in which participants were genotyped using either the UK BiLEVE Axiom Array or the UK Biobank Axiom Array, covering approximately 820,000 variants. Genotypes were centrally imputed using the 1000 Genomes Project and Haplotype Reference Consortium (HRC) reference panels, resulting in approximately 93 million variants per individual. Standard quality control procedures were applied, retaining autosomal variants with an imputation quality score > 0.3, a Hardy-Weinberg equilibrium p-value > 1 × 10^-5^, and an MAF > 0.05.

### Genetic correlations

Genome-wide genetic correlations across cancers were estimated using cleaned GWAS summary statistics via GNOVA [15], which accounts for sample overlaps between GWASs and performs pairwise correlation analyses. Bonferroni correction was applied to identify statistically significant cancer pairs.

Local genetic correlations were further evaluated using SUPERGNOVA [16], a method that provides stable estimates of regional genetic correlations [17]. The genome was partitioned into more than 2,000 approximately independent linkage disequilibrium (LD) blocks, and block-specific genetic correlations were computed for each cancer pair. Statistical significance was assessed using Bonferroni correction within each cancer pair. To identify shared correlated regions, we hypothesized that regions exhibiting local genetic correlations may contribute to shared risk across multiple cancers in a coordinated manner. Accordingly, we identified genomic regions having correlated cancer clusters involving at least three distinct cancer types. These regions were retained for downstream analyses.

Identified regions were annotated to characterize broader biological relevance in two complementary ways. First, we evaluated correlations with non-cancer phenotypes. GWAS summary statistics for 3,950 non-cancer traits, restricted to individuals of genetically inferred European ancestry, were obtained from the Neale Lab UKB analyses [18]. For each selected region, phenotypes with enriched local heritability (p-value < 0.05) were identified using LAVA [19]. Local genetic correlations between cancers in the region and the selected non-cancer phenotypes were then estimated using SUPERGNOVA, with significance assessed using Bonferroni correction. Second, variants within the regions of interest were extracted and annotated using the SNP2GENE function in FUMA [20] with the default positional mapping settings. Mapped genes were further examined using the GENE2FUNC module to identify previously reported associations in the GWAS Catalog. Through this approach, regional genetic correlations were linked to known variant-phenotype associations.

### PRS development and decomposition

To evaluate cross-cancer polygenic associations, we constructed genome-wide PRSs for each specific cancer. PRS weights were inferred from cleaned GWAS summary statistics using Summary statistics-based Dirichlet Process Regression (SDPR) [21], a Bayesian shrinkage method that does not require parameter tuning and has demonstrated stable predictive performance across multiple cancers [22]. SDPR employs a Dirichlet process prior on SNP effect-size variances, resulting in continuous shrinkage of effect estimates toward zero without explicitly assuming a fixed proportion of causal variants. Individual-level PRSs were calculated in the UKB participants as the weighted sum of risk alleles using PLINK 1.9 [23]. All PRSs were standardized to have a mean of zero and a standard deviation of one. Predictive performance was evaluated using the area under the receiver operating characteristic curve (AUC), and the statistical significance was assessed accordingly. Associations between PRSs and cancer outcomes were summarized using odds ratios (ORs).

To identify cancer pairs sharing polygenic risk, we conducted pairwise association analyses. For each cancer pair (i, j), cancer i was modeled as the outcome, and its association with the PRS for cancer j was evaluated using logistic regression, adjusting for the PRS for cancer i, age at recruitment, sex (except for sex-specific cancers), and the top 10 genetic principal components (PCs). The inclusion of PRS for cancer i was intended to evaluate whether the PRS for cancer j provided information on the risk of cancer i beyond that explained by the PRS for cancer i. This procedure was repeated across all cancer pairs, and statistically significant associations were identified. Because cancer comorbidity may be influenced by factors beyond shared common polygenic susceptibility, including rare variants, somatic alterations, and environmental exposures, we conducted sensitivity analyses excluding individuals diagnosed with both cancers to assess the robustness of the observed associations. Specifically, analyses were repeated among participants diagnosed with cancer i but not cancer j. Cancer pairs that remained significant after exclusion of comorbid cases were considered to share polygenic risk independent of clinical co-occurrence.

To investigate potential mechanisms underlying shared polygenic risk, we decomposed PRSs into functionally informed component PRSs using two complementary strategies. These approaches aimed to attribute across-cancer PRS associations to broader pleiotropic or biological processes rather than individual variants.

First, we applied our recently developed pleiotropy-decomposed PRS (PD-PRS) framework, which decomposed the genome-wide PRS into pleiotropy-informed components based on local genetic correlations [24]. Briefly, the genome was partitioned into approximately 2,000 independent LD blocks. Block-specific genetic correlations between cancers and 47 non-cancer phenotypes—grouped into 12 pleiotropy groups [25]—were estimated using SUPERGNOVA [16]. These groups were defined using domain knowledge of phenotype relationships, with their biological relevance further supported by significant genetic correlations among grouped phenotypes. Variants located within blocks showing significant correlations (p-value < 0.05) were assigned to the corresponding pleiotropy groups. For each pleiotropy group, a PD-PRS was constructed using variants assigned to that group and SDPR-derived genome-wide PRS weights, implemented with PLINK v1.9 [23]. An additional PD-PRS was defined using variants not assigned to any pleiotropy groups. In total, 13 PD-PRSs were generated for each cancer.

For each selected cancer pair, logistic regression models were fitted with cancer i (excluding individuals with cancer j) as the outcome and each PD-PRS for cancer j as the exposure, adjusting for the PRS of cancer i, age at recruitment, sex (except for sex-specific cancers), and the top 10 genetic PCs. To quantify the contribution of each pleiotropy group to the across-cancer association, we evaluated attenuation in explained variance using Nagelkerke’s R^2^. Specifically, a full model including the PRS for cancer j (Equation (1)) was compared with reduced models substituting either the PD-PRS (Equation (2)) or the remaining PD-PRS constructed using variants not included in the PD-PRS (Equation (3)).


$$\begin{array}{c} \begin{array}{c} Cancer_{i}\;\left(Excluding\;patients\;with\;cancer_{j}\right)\sim PRS_{cancer_{j}}+PRS_{cancer_{i}}+covariates \end{array} \end{array}$$ (1)

$$\begin{array}{c} \begin{array}{c} Cancer_{i}\;\left(Excluding\;patients\;with\;cancer_{j}\right)\sim PDPRS_{cancer_{j}}+PRS_{cancer_{i}}+covariates \end{array} \end{array}$$ (2)

$$\begin{array}{c} \begin{array}{c} Cancer_{i}\;\left(Excluding\;patients\;with\;cancer_{j}\right)\sim RemainingPDPRS_{cancer_{j}}+PRS_{cancer_{i}}+covariates \end{array} \end{array}$$ (3)

Nagelkerke's R^2^ was calculated for each model. The $\Delta R^{\text{2}}$ for excluding PD-PRS was calculated as the difference between R^2^ of equation (1) and of equation (3), and the $\Delta R^{\text{2}}$ for excluding remaining PD-PRS was the difference between R^2^ of equation (1) and of equation (2). The attenuation in R^2^ was computed as the difference between $\Delta R^{\text{2}}$ the values for PD-PRS and for the remaining PD-PRS. To test the null hypothesis that the across-cancer association cannot be attributed to the PD-PRS, a permutation test was conducted with 1,000 permutations while preserving the associations between the outcome, the PRS for cancer *i*, and covariates. Specifically, we first fitted the null model including the PRS for cancer *i* and all covariates and estimated the corresponding outcome probabilities. Permuted outcomes were then generated by resampling from these fitted probabilities while preserving the overall number of cases. The association between the PD-PRS and the permuted outcomes was subsequently evaluated to obtain the empirical null distribution.

Second, we decomposed PRSs into pathway-specific PRSs (PS-PRSs), which assigned SNPs to genes and then labelled with pre-defined pathways, to evaluate shared biological mechanisms between cancers. A total of 8,004 autosomal genes from 364 KEGG pathways [26] were mapped to genetic variants using positional mapping with a ± 10-kilobase window. For each pathway, a PS-PRS was calculated using pathway-specific variant sets and SDPR-derived weights, implemented in PLINK 1.9 [23]. Across-cancer associations for PS-PRSs were evaluated using logistic regression models analogous to those described above.

### Statistical analyses

All statistical analyses were conducted using R version 4.2, except where noted.

## Results

**Fig 1:**
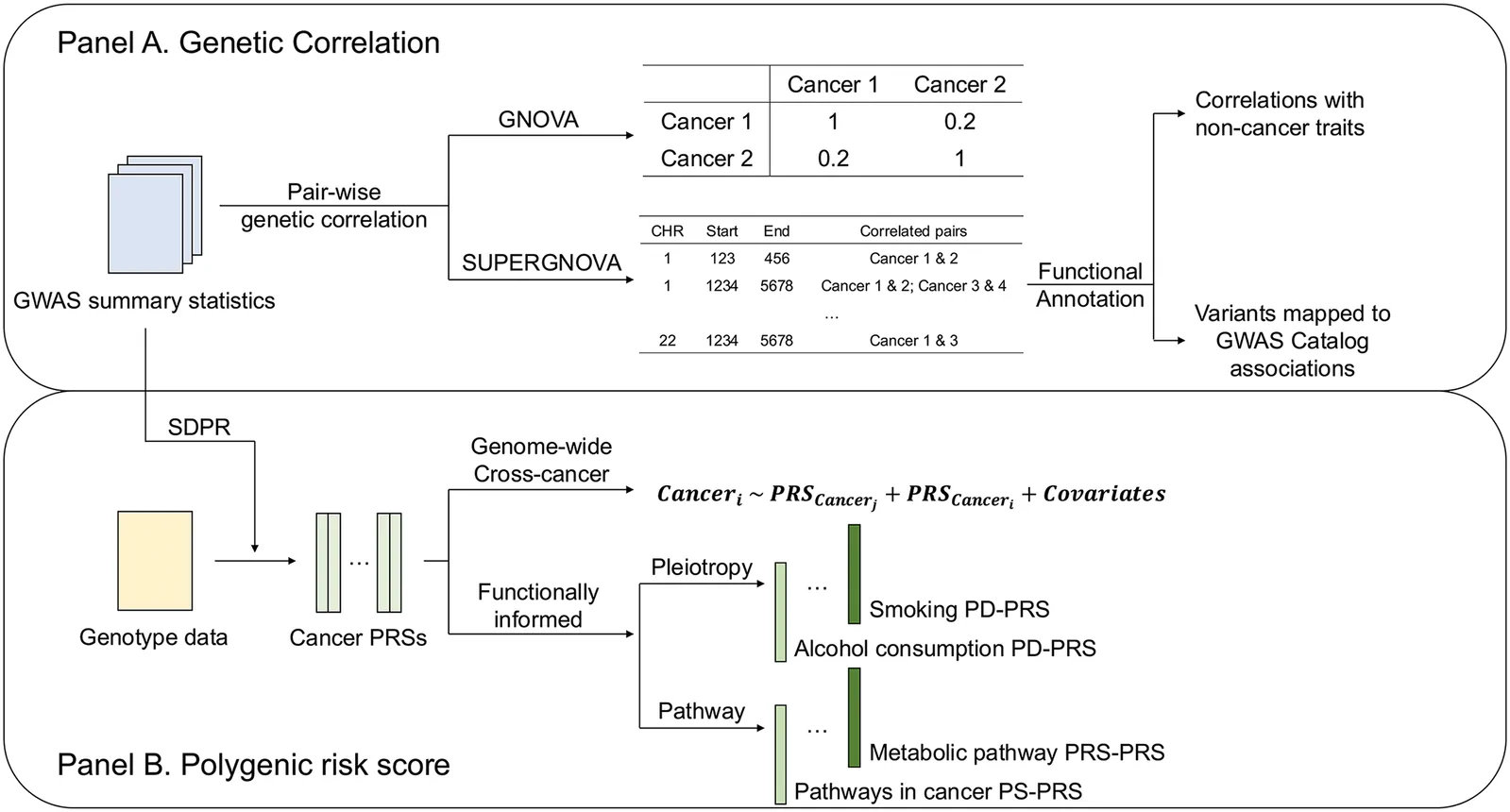
Overview of the study design. This figure summarizes the analytic framework used to characterize shared polygenic risk across cancers. Panel A shows the genetic correlation analyses based on GWAS summary statistics. Genome-wide genetic correlations were estimated pairwise using GNOVA, and local genetic correlations were calculated using SUPERGNOVA to identify genomic regions harboring correlated cancer clusters. Regions with shared signals were further annotated using genetic correlations with non-cancer traits and variant mappings from the GWAS Catalog. Panel B illustrates the PRS analyses. Genome-wide cancer PRSs were constructed using SDPR and evaluated in cross-cancer association analyses to identify cancer pairs with shared polygenic risk. For selected pairs, PRSs were further decomposed into pleiotropy-informed and pathway-specific components to assess functional enrichment of shared genetic risk.

An overview of the study design and analytic workflow is shown in Fig 1.

### GWAS summary statistics

We downloaded and cleaned GWAS summary statistics for 24 specific cancers and one pan-cancer phenotype. SNP-based heritability was estimated using LDSC [12]. Seventeen cancers showed significantly enriched heritability except for testis cancer, stomach cancer, oral cavity cancer, cervix uteri cancer, acute myeloid leukemia, nasopharyngeal cancer, chronic myeloid leukemia, and oropharyngeal cancer. Among the cancers with significant heritability estimates, prostate and breast cancers showed the highest estimates, h^2^ = 0.13 ± 0.02; and h^2^ = 0.13 ± 0.01, respectively, whereas acute lymphocytic leukemia (ALL) had the lowest (h^2^ = 0.002 ± 0.001) (S1 Table).

### Genetic correlations

**Fig 2:**
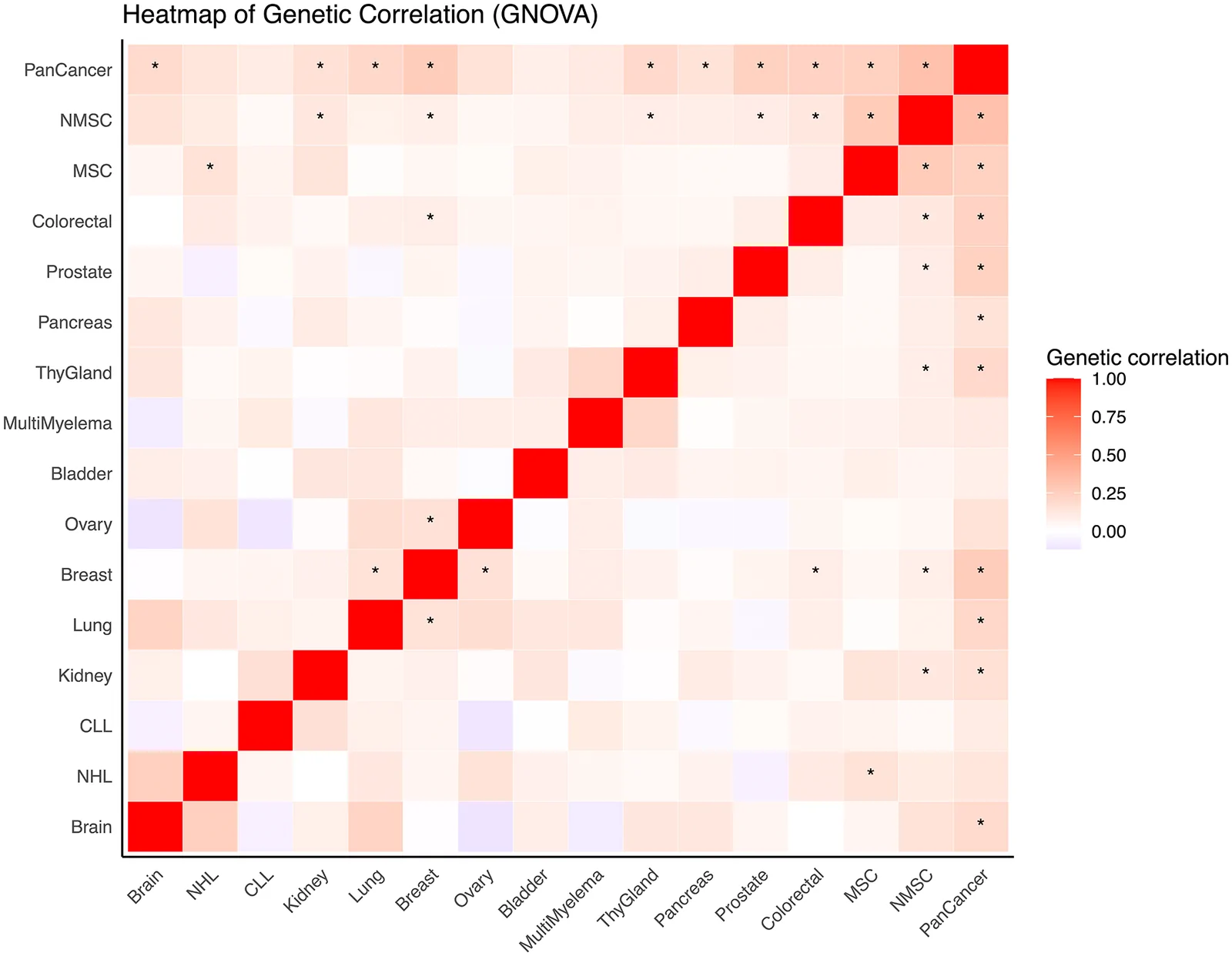
Genome-wide genetic correlations across cancers. Heatmap showing pairwise genome-wide genetic correlations among 15 specific cancers and one pan-cancer phenotype estimated using GNOVA. Each cell represents the genetic correlation coefficient between a pair of cancers, with red indicating positive correlations and blue indicating negative correlations. Asterisks denote correlations that remain significant after Bonferroni correction (p-value ≤ 4.17 × 10^-4^. Consistent with prior studies, significant genome-wide correlations were observed for a limited number of cancer pairs, with the pan-cancer phenotype and NMSC showing correlations with multiple specific cancers. Overall, genome-wide genetic sharing across cancers was modest.

Using GNOVA, we estimated pairwise genome-wide genetic correlations across 15 specific cancers and one pan-cancer phenotype. One more cancer, ALL, was excluded from this analysis due to negative heritability estimate in GNOVA. After Bonferroni correction for multiple testing, 20 cancer pairs showed significant correlations (p-value ≤ 4.17 × 10^-4^; Fig 2). Of these, 10 involved the pan-cancer phenotype, suggesting that some genetic risk factors contributing to individual cancers also influence aggregated cancer susceptibility. An additional 6 correlations involved non-melanoma skin cancer (NMSC). The remaining four correlated pairs were melanoma skin cancer (MSC)-non-Hodgkin lymphoma (NHL), breast-colorectal cancer, breast-lung cancer, and breast-ovarian cancers.

Furthermore, we evaluated local genetic correlations across 16 specific cancers. Following pair-specific Bonferroni correction, 82 unique genomic regions with significant local genetic correlations were identified across 66 cancer pairs (S3 Table). Among these pairs, nine also showed significant genome-wide genetic correlations. In contrast, the MSC-NHL pair, which was significantly genetically correlated, did not show any significant local genetic correlations. This discrepancy may reflect limited power to detect local genetic correlations for these cancers, a polygenic shared genetic architecture distributed across many genomic regions, or a combination of both factors. Of note, 57 cancer pairs showed significant local genetic correlations in the absence of genome-wide significance, suggesting that global correlation estimates may fail to capture regional genetic sharing.

Across the 82 identified regions, seven regions showed mutually correlated cancer clusters involving at least three cancer types (Table 1). One region on chromosome 2 (chr2:201572564–202829668; 2q33) was excluded because Chronic Lymphocytic Leukemia and Small Lymphocitis Leukemia (CLL) was a clinical subtype of Non-Hodgkin Lymphoma (NHL), and one region on chromosome 6 (chr6:32424108–32682443; 6p21) was excluded due to overlap with the major histocompatibility complex (MHC) since its extensive and complex LD structure can lead to unstable estimates and complicate the interpretation of local genetic correlation analyses. The remaining five regions were carried forward for functional annotation. Specifically, we examined local genetic correlations between cancers and non-cancer phenotypes and mapped variants within each region to known associations in the GWAS Catalog. Four of the five regions (excluding chr1:182294372–183796074; 1q25) showed at least one non-cancer phenotype that was significantly correlated with all cancers in the corresponding cluster (S4-S7 Tables). Variants within these regions were further mapped to gene sets using FUMA (S8 Table).

**Table 1:** Selected regions with mutually correlated cancer clusters.

Region (chr:start:end)	Cytoband	Mutually correlated pair(s)
1:182294372-183796074	1q25	Colorectal-NMSC-Prostate
2:201572564-202829668	2q33	CLL-NHL-NMSC
6:32424108-32682443	6p21	CLL-NHL-NMSC
		Bladder-NHL-NMSC
6:167178790-168548525	6q27	Lung-NHL-NMSC
8:128166556-128542444	8q24	Breast-Colorectal-Prostate
9:18660695-19129349	9p22	Lung-Prostate-ThyGland
11:63154309-66835194	11q12	Colorectal-NHL-NMSC
		Breast-Colorectal-NMSC

Chr: chromosome; NMSC: non-melanoma skin cancer; CLL: chronic lymphocytic leukemia and small lymphocitis leukemia; NHL: non-Hodgkin lymphoma; CML: chronic myeloid leukemia; ThyGland: thyroid gland cancer.

For the region chr6:167178790–168548525 (6q27), a mutually correlated cluster involving lung cancer, NHL, and NMSC was identified. All three cancers were significantly correlated with total protein levels in this region (S4 Table). Although total protein itself is not an established cancer risk factor, it reflects the combined abundance of circulating proteins and may capture underlying immune and inflammatory processes. This interpretation is supported by previous prospective proteomic studies demonstrating associations between numerous plasma proteins and the future risk of lung cancer and NHL [27]. In addition, variants within this region were enriched for 28 GWAS catalog traits, including thyroid function-related phenotypes such as thyroid-stimulating hormone levels and hyperthyroidism (S8 Table). This region has previously been associated with basal cell carcinoma [28], and may represent a locus with broader relevance to cancer risk.

In the chr8:128166556–128542444 (8q24) region, three cancers—breast cancer, colorectal cancer, and prostate cancer—showed shared correlations with six non-cancer phenotypes, including sibling history of prostate cancer (S5 Table). Variants were enriched for four cancers including urinary bladder carcinoma, breast carcinoma, CLL, and prostate carcinoma (S8 Table). The clustering of breast, colorectal, and prostate cancers at 8q24 is consistent with previous reports [8], further supporting the role of this region as a shared cancer susceptibility locus.

For chr9:18660695–19129349 (9p22), three cancers (lung cancer, prostate cancer, and thyroid gland cancer) were significantly correlated with 18 non-cancer phenotypes, including measures of lung function and blood cell counts (S6 Table). Variants in this region were mapped to 15 GWAS traits, including small-cell lung carcinoma (S8 Table). This region is an established risk region for ovarian cancer [29], and our results further extended it beyond a single cancer.

In the chr11:63154309–66835194 (11q12) region, four cancers (colorectal cancer, breast cancer, NHL, and NMSC) were correlated with 30 non-cancer phenotypes, notably obesity-related traits and blood cell indices (S7 Table), and variants were similarly enriched for obesity-related GWAS associations (S8 Table). This region has previously been associated with breast, prostate, and ovarian cancer risk [8,30]. Our findings further supported its role as a shared cancer susceptibility locus.

In contrast, for chr1:182294372–183796074 (1q25), no non-cancer phenotypes were significantly correlated across all cancers in the cluster. Variants in this region were mapped to seven GWAS traits, including chronotype-related phenotypes (S8 Table). Similar associations have been established with colorectal and prostate cancers previously [31,32], whereas our findings suggest a broader role for this locus in shared cancer susceptibility.

### PRS analyses

PRS weights were inferred for 16 specific cancers using SDPR, and individual-level PRSs were calculated for UKB participants of European ancestry. Discriminative performance was evaluated using AUC, and relative risk was summarized using ORs. Results are shown in S9 Table. The number of cases ranged from 124 for ALL to 25,666 for NMSC. Fifteen PRSs except ALL PRS showed AUCs and ORs significantly different from the null hypothesis (p-value < 0.05) and were retained for subsequent analysis.

Pairwise correlations among the 15 PRSs are shown in S1A Fig. Most PRSs were significantly correlated (p-value < 4.76 × 10^-4^), although the magnitudes of correlation were modest. Statistical significance was likely influenced by the large number of shared controls, whereas the small effect sizes reflected limited genome-wide genetic overlap across cancers. We further examined overlap among individuals in the top 10% of each cancer (S1B Fig). Consistent with the modest correlation coefficients, the proportion of shared high-risk individuals was approximately 10% for most cancer pairs. An exception was observed for NMSC and MSC, for which nearly 20% overlap was detected. Overall, these results indicate that genome-wide polygenic overlap across cancers is present but generally limited in magnitude.

We next assessed associations between 15 cancer-specific PRSs and specific cancer outcomes in the UKB using a two-step approach. In the first step, we tested cross-cancer associations by modeling each cancer outcome as a function of the PRS for another cancer, while adjusting for the target cancer's PRS. Among the 345 cancer-PRS pairs examined, 18 showed significant associations after Bonferroni correction (p-value < 1.45 × 10^-4^; Table 2). In the second step, we assessed whether these associations could be explained by clinical comorbidity by excluding individuals diagnosed with both cancers. After Bonferroni correction (p-value < 2.78 × 10^-3^), seven cancer-PRS pairs remained statistically significant (Table 2). These included associations between bladder cancer risk and the lung cancer PRS, as well as associations between lung cancer risk and the bladder cancer PRS. To further evaluate whether the identified shared polygenic risk can be fully attributed to smoking, we conducted stratified analyses for the bladder–lung cancer pair by smoking status. The association between bladder cancer and the lung cancer PRS remained significant among both ever and never smokers. In contrast, associations between lung cancer and the bladder cancer PRS were no longer significant in either stratum, but the effect estimates were similar to those observed in the pooled analysis (S10 Table), indicating potential loss of power in stratified analyses. Our results suggest that smoking-related mechanisms alone are unlikely to account for the observed genetic overlap between bladder and lung cancers. Based on these results, subsequent analyses focused on six cancer-PRS pairs: bladder cancer-lung cancer PRS, lung cancer-bladder cancer PRS, breast cancer-NMSC PRS, MSC-NMSC PRS, NMSC-CLL PRS, and NMSC-lung cancer PRS. Because CLL is a clinical subtype of non-Hodgkin lymphoma, the NMSC-CLL pair was excluded from further analyses.

**Table 2:** Significant cross-cancer associations for genome-wide PRSs.

		Step 1		Step 2	
Cancer	PRS	OR (95% CI)	P-value	OR (95% CI)	P-value
**Bladder**	**Lung**	**1.09 (1.06-1.12)**	**3.03E-08**	**1.08 (1.05-1.11)**	**6.93E-07**
Bladder	Prostate	1.07 (1.04-1.1)	1.78E-05	0.98 (0.95-1.01)	1.77E-01
**Breast**	**NMSC**	**1.06 (1.04-1.07)**	**5.99E-14**	**1.03 (1.01-1.04)**	**2.36E-04**
**CLL**	**NHL**	**1.16 (1.09-1.22)**	**3.39E-07**	**1.13 (1.07-1.2)**	**4.31E-05**
Colorectal	Breast	1.04 (1.02-1.06)	1.44E-04	1.01 (0.99-1.03)	2.08E-01
Colorectal	Prostate	1.05 (1.03-1.07)	1.14E-05	1.01 (0.99-1.03)	5.33E-01
Kidney	Breast	1.08 (1.04-1.12)	1.12E-04	1.06 (1.02-1.1)	4.02E-03
Kidney	NMSC	1.09 (1.05-1.13)	1.33E-05	1.05 (1.01-1.09)	1.43E-02
**Lung**	**Bladder**	**1.05 (1.02-1.08)**	**1.39E-04**	**1.04 (1.02-1.07)**	**1.12E-03**
Lung	NMSC	1.06 (1.03-1.09)	7.37E-06	1.04 (1.01-1.06)	8.69E-03
**MSC**	**NMSC**	**1.17 (1.14-1.21)**	**1.16E-24**	**1.09 (1.06-1.13)**	**6.03E-07**
NMSC	Breast	1.03 (1.02-1.05)	3.49E-07	1.01 (0.99-1.02)	4.51E-01
**NMSC**	**CLL**	**1.04 (1.03-1.06)**	**3.49E-10**	**1.04 (1.02-1.05)**	**5.85E-08**
**NMSC**	**Lung**	**1.05 (1.03-1.06)**	**6.69E-12**	**1.04 (1.03-1.06)**	**4.02E-10**
NMSC	Prostate	1.03 (1.01-1.04)	7.04E-05	0.98 (0.97-1)	1.34E-02
Ovary	Breast	1.12 (1.08-1.17)	5.64E-08	1.07 (1.02-1.12)	3.44E-03
Prostate	Colorectal	1.04 (1.02-1.06)	3.30E-05	1.03 (1.01-1.05)	3.00E-03
Prostate	NMSC	1.05 (1.03-1.07)	7.49E-08	1.01 (0.99-1.03)	4.18E-01

PRS: polygenic risk score; OR: odds ratio; CI: confidence interval.

**Bold: significant after Bonferroni correction in step 2 (p-value < 2.78 × 10** ^**-3**^ **).**

PRSs involved in the six selected cancer pairs—lung cancer PRS, bladder cancer PRS, NMSC PRS, and CLL PRS—were involved for the following decompositions. Specifically, we applied two decomposition frameworks to the four selected genome-wide PRSs to further characterize sources of shared polygenic risk. Specifically, PRSs were decomposed into pleiotropy-informed PD-PRSs and pathway-specific PS-PRSs, and cross-cancer associations were evaluated using these component scores.

#### PD-PRSs.

The genome-wide PRSs for bladder cancer, lung cancer, NMSC, and CLL were decomposed into 14 PD-PRSs, with the number of SNPs in each component summarized in S2 Fig. For all three cancers, the largest proportion of variants was assigned to the other PD-PRS, which comprised variants located in genomic regions showing no detectable genetic correlation with the 47 non-cancer phenotypes. Associations between PD-PRSs and their corresponding cancers were evaluated using logistic regression models with covariate adjustment (S3 Fig). All PD-PRSs for lung cancer and NMSC were significantly associated with their respective cancers after Bonferroni correction (p-value < 8.93 × 10^-4^; S3C-S3D Fig). For bladder cancer, associations with alcohol consumption- and hypertension-related PD-PRSs failed to reach significance (S3A Fig). For CLL, two PD-PRSs—alcohol consumption and chronic kidney disease—were not significantly associated and were excluded from the following analyses (S3B Fig). Despite containing the largest number of variants, the other PD-PRS did not consistently yield the strongest associations with cancer risk. Instead, higher ORs were often observed for PD-PRSs corresponding to specific pleiotropy groups, suggesting that cancer-associated genetic risk may be enriched invariants shared with non-cancer phenotypes. For bladder cancer, the neuropsychiatric disease PD-PRS showed the strongest association (OR = 1.29, 95% CI: 1.26-1.33), implicating enrichment of related function in the development of bladder cancer. For CLL, the autoimmune disease PD-PRS showed the strongest association (OR = 1.45, 95% CI: 1.38-1.54), supporting an immune-related component of genetic susceptibility. For lung cancer, the diabetes PD-PRS exhibited the largest effect size (OR = 1.27, 95% CI: 1.23-1.30), consistent with evidence linking metabolic dysfunction, insulin resistance, and diabetes-related pathways to cancer development and progression [33]. For NMSC, the neuropsychiatric disease PD-PRS showed the strongest association (OR = 1.46, 95% CI: 1.44–1.48), suggesting that genetic components enriched for neuropsychiatric disease may also contribute to NMSC susceptibility. Although the underlying mechanisms remain unclear, previous studies have reported shared genetic architecture between neurological disorders such as Parkinson’s disease and skin cancers, potentially reflecting overlap in pigmentation biology, neural crest development, DNA damage response, and immune regulation [34].

**Fig 3:**
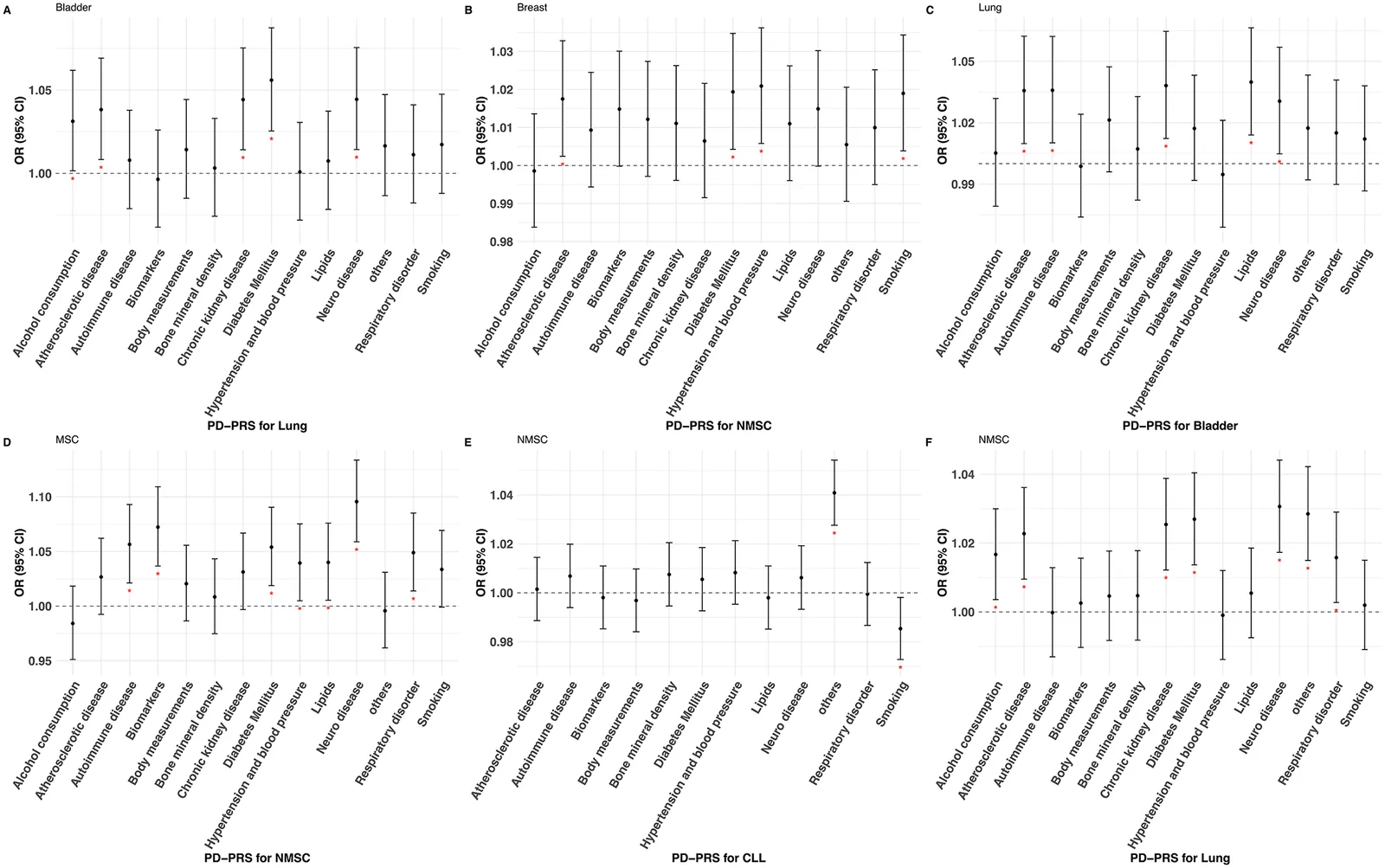
Cross-cancer associations of PD-PRS. ORs and 95% confidence intervals for associations between PD-PRSs and cancer outcomes after excluding individuals with comorbid cancers are shown for six cancer pairs of interest. Each panel corresponds to a target cancer outcome, and PD-PRSs were derived from the genome-wide PRS of the paired cancer. Asterisks indicate nominally significant associations (p-value < 0.05). Across pairs, only a subset of PD-PRS components showed significant associations, indicating that cross-cancer polygenic overlap is concentrated in specific pleiotropy-defined trait domains rather than uniformly distributed across the genome.

Further, we analyzed cross-cancer associations (excluding comorbidity) using PD-PRSs for the six cancer pairs identified in prior analyses. Among 82 tested associations, 30 were statistically significant (p-value < 0.05; Fig 3 and S11 Table). For the bladder-lung cancer pair, the diabetes PD-PRS for lung cancer showed the strongest association with bladder cancer risk (OR = 1.06, 95% CI: 1.03-1.09), consistent with its prominent role in lung cancer susceptibility. While lung cancer-bladder cancer PRS pair, the diabetes PD-PRS for bladder cancer was not associated with lung cancer risk (OR = 1.02, 95% CI: 0.99-1.04), and the lipids PD-PRS showed the strongest association (OR = 1.04, 95% CI: 1.01-1.07). For the breast-NMSC pair, four PD-PRSs showed significant associations with comparable effect sizes. Notably, the neuropsychiatric PD-PRS, which showed the strongest association with NMSC, was not associated with breast cancer but instead showed its strongest association with MSC, suggesting cancer-specific patterns of pleiotropy. For the NMSC-CLL pair, two CLL PD-PRSs—others and smoking PD-PRSs—were significantly associated with NMSC, although the association with the smoking PD-PRS was inverse (OR = 0.99, 95% CI: 0.97-1). In addition, seven lung cancer PD-PRSs, including diabetes PD-PRS, were significantly associated with NMSC risk. Together, these findings suggested that cross-cancer PRS associations are driven by specific pleiotropic components rather than by uniform genome-wide effects.

**Fig 4:**
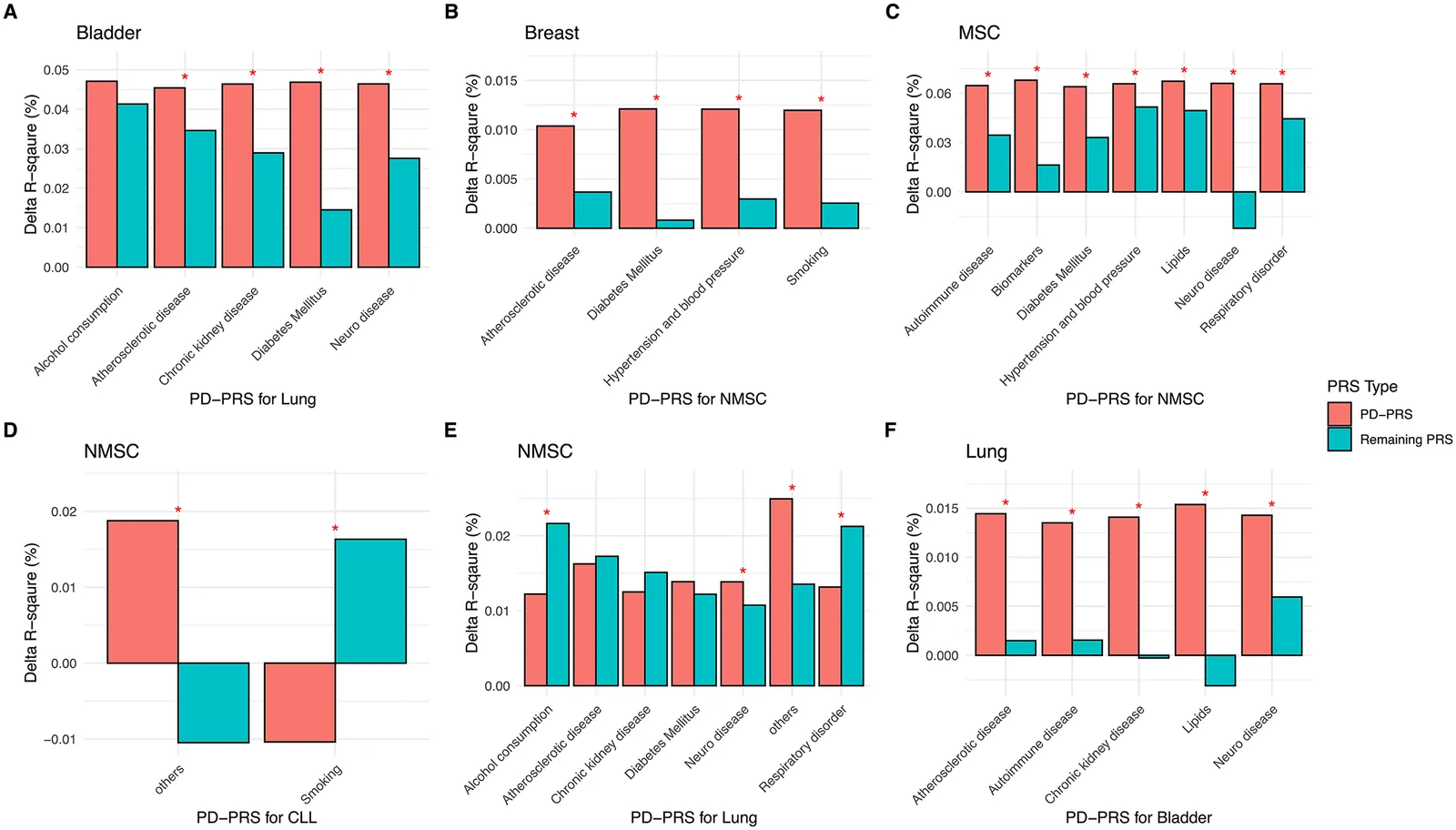
Attenuation of explained variance (${\Delta R}^{2}$) for PD-PRSs. For each selected cancer pair, the proportion of variance explained $\Delta R^{\text{2}}$ by the PD-PRS (red) was compared with that explained by the corresponding remaining PRS (blue). Each panel represents one selected cancer-PRS pair. Asterisks indicate PD-PRS components for which the difference in $\Delta R^{\text{2}}$ between the PD-PRS and remaining PRS was statistically significant based on the permutation testing (1,000 permutations). Although the overall variance explained by cross-cancer PRSs was modest, selected PD-PRS components accounted for a larger share of the explained variance, indicating enrichment of shared polygenic risk within specific functions.

To formally assess whether cross-cancer associations could be attributed to specific PD-PRSs, we evaluated attenuation in explained variance using differences in Nagelkerke’s $R^{\text{2}}$. Among the 30 significant associations, 26 showed significantly different $\Delta R^{\text{2}}$ values between the PD-PRS and the remaining PRS (permutation p-value < 0.05; Fig 4). In 23 of these associations, the PD-PRS explained a larger proportion of the variance than the remaining PRS, suggesting enrichment of shared genetic risk within specific pleiotropy components. For bladder cancer risk, lung cancer PD-PRSs related to atherosclerotic disease, chronic kidney disease, diabetes, and neuropsychiatric disease showed significantly greater $\Delta R^{\text{2}}$, while for lung cancer, all five associated bladder cancer PD-PRSs related to atherosclerotic disease, autoimmune disease, chronic kidney disease, lipids, and neuropsychiatric disease showed significantly greater $\Delta R^{\text{2}}.$ All four PD-PRSs for NMSC associated with breast cancer, as well as all seven PD-PRSs for NMSC associated with MSC, demonstrated larger $\Delta R^{\text{2}}$ values. In contrast, for the NMSC-CLL pair, only the other PD-PRS showed greater variance explained, suggesting that additional or uncharacterized mechanisms may contribute to this association. Overall, decomposing genome-wide cancer PRSs into pleiotropy-informed components enabled quantification of relative contributions of specific phenotype groups to cross-cancer associations.

#### PS-PRSs.

To examine biologically informed polygenic sharing, variants included in the PRSs for bladder cancer, CLL, lung cancer, and NMSC were mapped to 8,003 autosomal genes and subsequently assigned to 364 KEGG pathways. The number of variants contributing to each PS-PRS is summarized in S12 Table. The pathways containing the largest numbers of variants were metabolic pathways, pathways in cancer, and pathways related to neurodegenerative diseases. PS-PRSs were calculated for UKB participants of European ancestry as weighted sums of pathway-specific variants, using SDPR-derived genome-wide weights.

**Fig 5:**
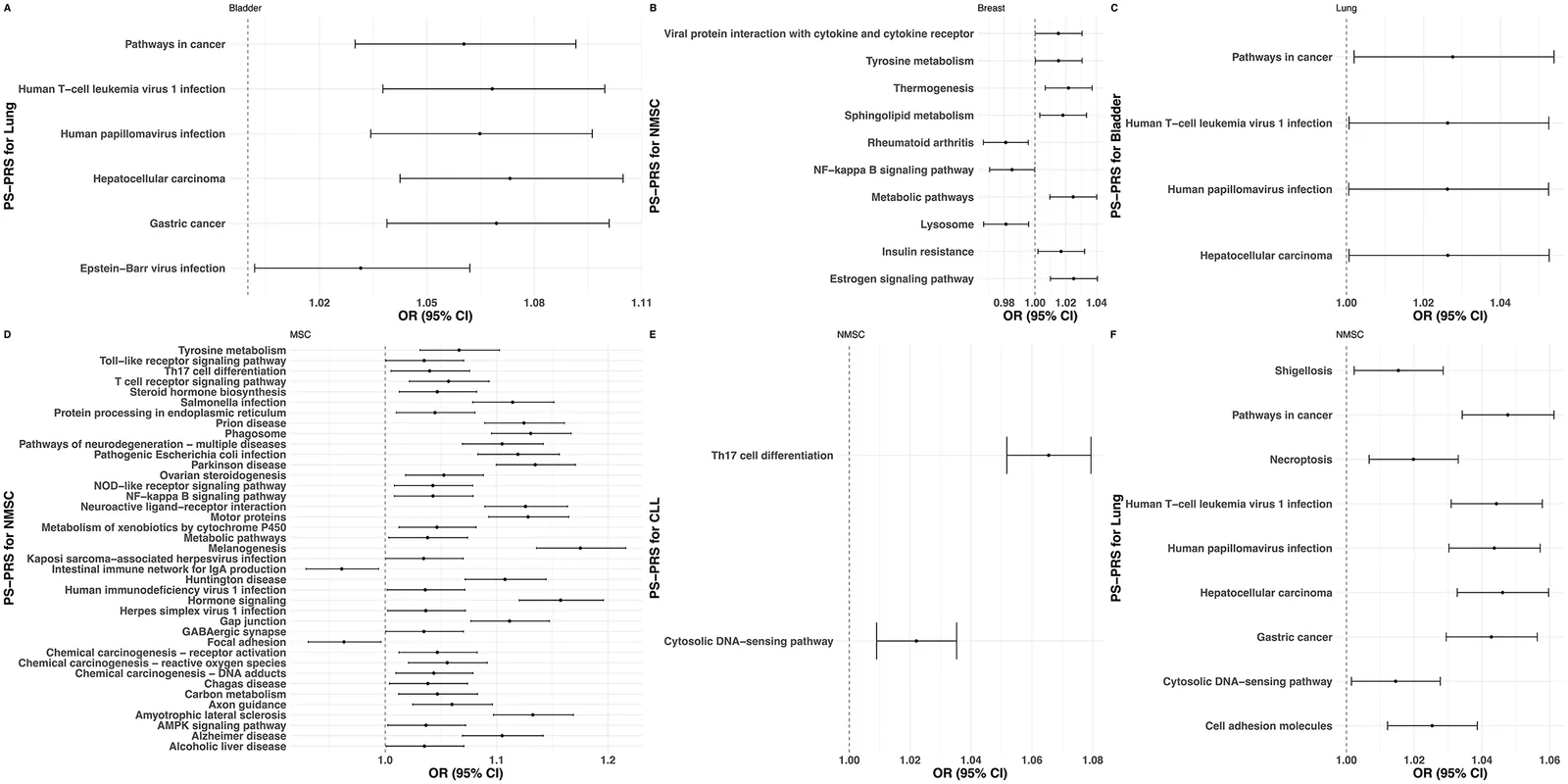
Significant cross-cancer associations of PS-PRS. ORs and 95% confidence intervals for 70 significant associations (p-value < 0.05) between PS-PRSs and cancer outcomes after excluding individuals with comorbid cancers are shown for five pairs of interest. Each panel corresponds to a target cancer outcome, and PS-PRSs were derived from the genome-wide PRS of the paired cancer. Across all PS-PRSs, only subset of PS-PRSs showed significant cross-cancer associations, suggesting the genetic overlap between cancers might converge to specific functional pathways.

Associations between PS-PRSs and their corresponding cancers were evaluated using logistic regression. After Bonferroni correction, 258 PS-PRSs were significantly associated with their target cancers (p-value < 3.42 × 10^-5^), including 21 for bladder cancer, 49 for CLL, 31 for lung cancer, and 174 for NMSC (S13 Table). We next evaluated cross-cancer PS-PRS associations for the six cancer pairs identified in prior analyses, excluding individuals with comorbid diagnoses. Using a nominal significance threshold (p-value < 0.05), 70 significant cross-cancer associations were identified (Fig 5 and S14 Table).

To assess overlap among pathways, we examined gene sharing using UpSet plots and Jaccard similarity indices (S4 Fig). For the bladder-lung cancer pair, six lung cancer PS-PRSs showed significant associations with bladder cancer risk. Gene overlap across these pathways was limited, except for the gastric cancer pathway (S4A–S4B Fig). These pathways are broadly clustered into cancer-related and infection-related categories, indicating that polygenic overlap between bladder and lung cancer is distributed across multiple functional groups rather than driven by a single shared pathway. Similar pathways were found for associations between bladder cancer PS-PRS and lung cancer risk (S4E–S4F Fig), further supporting the roles of these pathways in genetic overlap for these two cancers. For the NMSC-MSC pair, 39 NMSC PS-PRSs were significantly associated with MSC. Although gene overlap across pathways was generally limited, clusters of pathways related to neurodegenerative diseases and metabolic processes were observed, indicating convergence at the functional category level rather than at the gene level (S4G–S4H Fig). Only two CLL PS-PRSs were significantly associated with NMSC. These pathways did not share genes (S4I–S4J Fig), suggesting that shared polygenic risk between CLL and NMSC may arise from distinct biological processes rather than common pathway-level effects. For the NMSC–lung cancer pair, nine lung cancer PS-PRSs were significantly associated with NMSC (S4K–S4L Fig). While gene overlap across pathways remained limited, several functional clusters were evident, including infection-related immune pathways, cancer-related pathways, and other immune-associated processes.

Overall, PS-PRS analyses indicate that cross-cancer polygenic overlap is distributed across multiple biological pathways with limited gene-level overlap. These results suggest that shared genetic susceptibility across cancers reflects convergence within broader functional categories rather than dependence on specific genes or pathways.

## Discussion

In this study, we characterized shared genetic architecture across cancers by integrating genetic correlation analyses with PRS-based approaches. Local genetic correlation analyses revealed clusters of multiple cancers sharing regional genetic susceptibility that was not apparent from genome-wide correlations alone. Complementary cross-cancer PRS analyses identified a limited number of cancer pairs with shared polygenic risk. Decomposition of genome-wide PRSs into pleiotropy- and pathway-informed components further indicated that cross-cancer associations are driven by structured enrichment of shared genetic effects across specific non-cancer trait groups and biological pathways, rather than by uniform genome-wide overlap. To conclude, we confirmed limited average genetic sharing at the genome-wide scale across cancers and found that these signals were enriched for specific functions, such as immune-related pathways.

Our observation of a limited number of correlated cancer pairs and cross-cancer PRS associations reinforces prior evidence that polygenic risk sharing across cancers is generally modest [6–9]. Importantly, the cancer pairs identified by our analyses recapitulate well-established relationships among cancers, supporting the validity of our analytic framework. For example, genome-wide genetic correlations between breast cancer and colorectal cancer, as well as between breast cancer and lung cancer, have been reported previously [7]. Similarly, the association between bladder cancer risk and the lung cancer PRS identified in our study is consistent with findings from Sampson et al. (2015), which reported both a significant genetic correlation between these cancers and cross-cancer PRS associations driven by a subset of risk variants [6]. We also observed a unidirectional association in which the PRS for CLL was associated with NMSC, whereas the NMSC PRS was not associated with CLL, a pattern consistent with prior work [35]. Together, these findings confirmed the limited genetic sharing globally across cancers and advocated regional and functional analyses.

By integrating local genetic correlation analyses with functionally informed PRS decomposition, we demonstrate that shared polygenic risk across cancers, while limited in magnitude, converges on specific functions. Such a regional and polygenic organization is not apparent from genome-wide correlation analyses alone. For example, beyond global genetic correlations, we identified a multi-cancer regional cluster on chromosome 8 (chr8:128166556–128542444; 8q24) shared by breast, colorectal, and prostate cancers. This region corresponds to 8q24, a well-established cancer susceptibility locus that can be missed by genome-wide correlation metrics despite its relevance across cancer types [7]. By identifying 8q24 through local correlation clustering and annotating it with non-cancer traits, our analysis places this locus within a broader framework of shared regional genetic susceptibility across cancers. Consistent with prior biological knowledge, this region contains multiple long non-coding RNAs and has been linked to cancer risk through regulation of the proto-oncogene *MYC* [36]. In addition, cancers within this cluster showed shared genetic correlations with hypertension-related traits, aligning with previous evidence linking MYC to blood pressure regulation [37].

Decomposition of genome-wide PRSs into pleiotropy-informed and pathway-specific components further extends current understanding of cross-cancer PRS associations. Specifically, our PD-PRS analyses indicate that the shared polygenic signal between bladder and lung cancers is enriched for genetic components associated with atherosclerotic diseases and neuropsychiatric phenotypes (Fig 3). Complementary PS-PRS analyses highlight enrichment in immune- and virus-related pathways (Fig 5). Although these findings do not implicate a single causal mechanism, they suggest that the shared genetic susceptibility between bladder and lung cancers may arise from interconnected biological processes involving multiple functions. These findings provide a structured framework for interpreting cross-cancer PRS associations and suggest that shared polygenic risk is concentrated within specific functional domains rather than distributed uniformly across the genome.

We note several limitations of our study. First, our analyses focused on polygenic risk derived from common variants and did not incorporate rare variants or somatic mutations, which play important roles in cancer susceptibility and progression. We also did not evaluate variant-level pleiotropy, which may provide complementary insight into shared cancer risk. Second, some established pleiotropic cancer loci may not have been detected because of limitations of the predefined regional partitioning, as illustrated by the *TERT* gene being split across two adjacent regions (chr5:10,056-1,267,356 and chr5:1,270,983-1,762,678), or because of limited power and heterogeneity in the input GWAS summary statistics, as observed for the *ABO* gene. Third, this study was designed to be hypothesis-generating, and independent validation will be necessary to confirm the identified patterns of shared genetic architecture. Fourth, both genetic correlations and cross-cancer PRS associations were modest in magnitude, indicating that shared polygenic risk explains only a limited proportion of cancer variance. Fifth, cancer phenotypes included in this study may be heterogeneous with respect to tumor subtype and clinical characteristics, which could attenuate cancer-specific patterns of shared genetic architecture. In addition, statistical power varied across cancer types because of substantial differences in sample size, potentially limiting the detection of shared genetic signals for less prevalent cancers. Furthermore, the cancer GWASs and UKB analyses included prevalent cancer cases diagnosed before genotyping. Because germline genetic risk is fixed at birth, this design is unlikely to substantially affect the estimated inherited susceptibility. Nevertheless, future studies restricted to incident cancer cases would be valuable to further evaluate the robustness of our findings. Additionally, differences in genetic ancestry across the included GWASs, particularly between FinnGen and other European-ancestry studies, may have influenced the estimated genetic correlations. Future studies using more ancestrally homogeneous datasets will be valuable for validating and refining these estimates. Sixth, the use of cancer-free controls in several source GWASs may have modestly influenced estimates of cross-cancer genetic correlation because controls were depleted of risk alleles associated with other cancer types. Future studies using alternative control definitions may help further evaluate the impact of this design choice on pleiotropy estimates. Additionally, the exclusion of individuals with multiple cancer diagnoses in sensitivity analyses may have reduced the ability to detect certain pleiotropic effects, as these individuals could be enriched for shared genetic susceptibility across cancers. Of note, the relatively modest sample sizes of several source GWASs may have limited statistical power to detect shared genetic signals, particularly for less common cancers. Finally, all analyses were restricted to individuals of European ancestry, limiting generalizability to other populations.

In conclusion, our results show that shared genetic architecture across cancers is generally modest at the genome-wide level but becomes apparent when examined at regional scales. Local genetic correlation analyses identified multi-cancer genomic regions that are not captured by global correlations, while cross-cancer PRS analyses highlighted a limited set of cancer pairs with polygenic overlap. Annotations of correlated regions and PRS further indicated that this shared risk is structured and enriched in specific genetic domains rather than uniformly distributed across the genome. Together, these findings refine current understanding of cross-cancer genetic sharing and provide an integrative framework for studying how shared polygenic susceptibility manifests across cancer types.

## Supporting Information

S1 Table GWAS summary statistics. (XLSX)

S2 Table Cancer codes in the UK Biobank. (XLSX)

S3 Table Significant local genetic correlations. (XLSX)

S4 Table Correlations with non-cancer phenotypes in chr6:167178790-168548525. (XLSX)

S5 Table Correlations with non-cancer phenotypes in chr8:128166556–128542444. (XLSX)

S6 Table Correlations with non-cancer phenotypes in chr9:18660695–19129349. (XLSX)

S7 Table Correlations with non-cancer phenotypes in chr11:63154309–66835194. (XLSX)

S8 Table GWAS Catalog associations for selected regions. (XLSX)

S9 Table Associations between PRS and target cancers. (XLSX)

S10 Table Associations between bladder-lung cancer pair stratified by smoking status. (XLSX)

S11 Table Cross-cancer associations of PD-PRSs. (XLSX)

S12 Table Number of SNPs included in PS-PRSs. (XLSX)

S13 Table Associations between PS-PRSs and target cancers. (XLSX)

S14 Table Cross-cancer associations of PS-PRSs. (XLSX)

S1 Fig Correlations and shared high-risk proportions across PRSs. (A) Heatmap of pairwise correlations among 15 cancer PRSs. Each cell represents the Pearson correlation coefficient, with red indicating positive correlations and blue indicating negative correlations. Statistically significant correlations (p-value < 0.05) are marked with asterisks in the upper triangle, while correlation coefficients are shown in the lower triangle. Although many PRSs were significantly correlated, the magnitude of correlations was generally modest. (B) Heatmap of the proportion of shared high-risk participants (top 10% PRS) across 15 cancer PRSs. Each cell represents the proportion of individuals classified as high risk for both PRSs, with numeric values displayed in the lower triangle.
(TIF)

S2 Fig Number of SNPs included in PD-PRSs. This figure displays the number of SNPs included in each of the 14 PD-PRSs for bladder cancer (red), CLL (green), lung cancer (blue), and NMSC (purple). Each bar corresponds to one PD-PRS, and bar height indicates the number of SNPs assigned to that component. Across all three cancers, the other PD-PRS contained the largest number of SNPs, followed by the diabetes-related PD-PRS.
(TIF)

S3 Fig Associations between PD-PRSs and target cancers. This figure shows the ORs and 95% CIs for associations between PD-PRSs and their corresponding target cancers for (A) bladder cancer, (B) CLL, (C) lung cancer, and (D) NMSC. Associations that remained significant after Bonferroni correction (p-value < 8.93 × 10^-4^) are indicated with asterisks. All PD-PRSs were significantly associated with the corresponding target cancer outcome, with exception of the alcohol consumption- and hypertension and blood pressure-related PD-PRSs for bladder cancer and alcohol consumption– and chronic kidney disease–related PD-PRSs for CLL.
(TIF)

S4 Fig Gene overlap across PS-PRSs. This figure summarizes gene overlap across PS-PRSs using UpSet plots (A, C, E, G, I, K) and pairwise Jaccard similarity heatmaps (B, D, F, H, J, L) for six cancer pairs: (A-B) lung cancer PS-PRSs associated with bladder cancer; (C-D) NMSC PS-PRSs associated with breast cancer; (E-F) bladder cancer PS-PRSs associated with lung cancer; (G-H)NMSC PS-PRSs associated with MSC; (I-J) CLL PS-PRSs associated with NMSC; and (K-L) lung cancer PS-PRSs associated with NMSC. Across all pairs, overlap of genes across PS-PRSs was limited, and Jaccard similarity coefficients were generally low, indicating that cross-cancer polygenic overlap is distributed across distinct pathway-level gene sets rather than driven by a shared set of individual genes.
(TIF)
