# Supplementary material for: Identifying shared polygenic risk across cancers
Source: PLoS Genet. 2026 Sep 18;22(9):e1012308. doi: 10.1371/journal.pgen.1012308 (PMC13600620; doi:10.1371/journal.pgen.1012308)
Supplement: Supplementary file 1 [file pgen.1012308.s019.docx]

**Journal Requirements:**

1) Please ensure that the CRediT author contributions listed for every co-author are completed accurately and in full.
At this stage, the following Authors/Authors require contributions: jiaqi hu. Please ensure that the full contributions of each author are acknowledged in the "Add/Edit/Remove Authors" section of our submission form.
The list of CRediT author contributions may be found here: <https://journals.plos.org/plosgenetics/s/authorship#loc-author-contributions>

2) Please provide an Author Summary. This should appear in your manuscript between the Abstract (if applicable) and the Introduction, and should be 150-200 words long. The aim should be to make your findings accessible to a wide audience that includes both scientists and non-scientists. Sample summaries can be found on our website under Submission Guidelines:
<https://journals.plos.org/plosgenetics/s/submission-guidelines#loc-parts-of-a-submission>

3) Please upload all main figures as separate Figure files in .tif or .eps format. For more information about how to convert and format your figure files please see our guidelines: 
<https://journals.plos.org/plosgenetics/s/figures>

4) We have noticed that you have uploaded Supporting Information files, but you have not included a list of legends. Please add a full list of legends for your Supporting Information files after the references list.

5) Thank you for stating that "The summary-level data (e.g. PRS weights) will be available on the PGS catalog (<https://www.pgscatalog.org/>) once published." We strongly recommend all authors deposit their data before acceptance, as the process can be lengthy and hold up publication timelines. Please note that, though access restrictions are acceptable now, your entire minimal dataset will need to be made freely accessible if your manuscript is accepted for publication. This policy applies to all data except where public deposition would breach compliance with the protocol approved by your research ethics board.

6) Your current Financial Disclosure states, "The author(s) received no specific funding for this work.".
However, your funding information on the submission form indicates receiving funds.Please ensure that the funders and grant numbers match between the Financial Disclosure field and the Funding Information tab in your submission form. Note that the funders must be provided in the same order in both places as well.

Please amend your detailed Financial Disclosure statement. This is published with the article. It must therefore be completed in full sentences and contain the exact wording you wish to be published.
1) Please clarify all sources of financial support for your study. List the grants, grant numbers, and organizations that funded your study, including funding received from your institution. Please note that suppliers of material support, including research materials, should be recognized in the Acknowledgements section rather than in the Financial Disclosure
2) State the initials, alongside each funding source, of each author to receive each grant. For example: "This work was supported by the National Institutes of Health (####### to AM; ###### to CJ) and the National Science Foundation (###### to AM)."
3) State what role the funders took in the study. If the funders had no role in your study, please state: "The funders had no role in study design, data collection and analysis, decision to publish, or preparation of the manuscript."
4) If any authors received a salary from any of your funders, please state which authors and which funders.

7) Please revise your current Competing Interest statement to the standard "The authors have declared that no competing interests exist."

Note: If the reviewer comments include a recommendation to cite specific previously published works, please review and evaluate these publications to determine whether they are relevant and should be cited. There is no requirement to cite these works unless the editor has indicated otherwise.

**Reviewers' comments:**

Reviewer's Responses to Questions

Reviewer #1: This paper uses publicly available summary genome-wide association data and individual-level data from the UK Biobank to assess the genetic correlations between cancers and decompose these correlations into putative functional components. The top-line findings are in line with previous studies [PMIDs 36929942 30683880 26464424 27432226]: although the overall genetic correlations between cancers are weak, there are regions with stronger correlations among multiple cancers (e.g. TERT, HLA, 8q24, ABO, others discussed below).The authors use these local correlations between cancers and correlations between cancers and other traits to assess the mechanisms driving these correlations. These analyses take an approach that has previously been used to understand the polygenic contribution to individual traits (including at least one cancer) [41315867 38374256 38443691 <https://www.medrxiv.org/content/10.1101/2025.05.15.25327708v1>] and apply it to multiple traits.

Response: We thank the reviewer for highlighting studies related to our work. We agree that prior work has applied local genetic correlation analyses to investigate shared polygenic architecture across traits and cancers. Our study builds upon these approaches by integrating local genetic correlation analyses with decomposed partial PRSs to characterize pleiotropic genetic components across cancers at both the locus and individual levels. To our knowledge, this combined framework has not been previously applied in the context of cross-cancer pleiotropy analysis.

The paper is hard to follow in some places and could use more details in the methods and results sections (specifics below). Some of the authors’ interpretations of their results are “too little” (e.g. tautological restatements of the empirical results) and some are “too much,” latching on to one possible explanation for the results, without considering alternatives (again, specifics below). There are also some analytic choices that I would not have made, and I didn’t find the authors’ justification compelling: notably, the focus on analyses removing individuals with multiple cancers, which shifts the focus away from shared genetic contributions to distinct genetic and environmental contributions.

Response: We thank the reviewer for the thoughtful and detailed comments. We appreciate the concerns regarding clarity of presentation, interpretation of findings, and the rationale underlying several analytic choices. In response, we have substantially revised the Methods and Results sections to improve readability and provide additional methodological details and clarifications throughout the manuscript. We also revised portions of the Discussion to provide more balanced interpretations of the findings and to better acknowledge alternative explanations where appropriate.

Regarding the analyses excluding individuals with multiple cancers, our intention was not to shift the focus away from shared genetic contributions, but rather to evaluate the robustness of the observed signals in analyses less influenced by cancer comorbidity patterns. We have further clarified the rationale for these analyses and discussed their limitations and interpretation in the revised manuscript.

But the biggest limitation of this paper is the limited power in most of the input GWAS and the UK Biobank.The GWAS summary statistics for 12 of the 24 cancers they considered come from a single biobank, FinnGen, with small case numbers (median of 1,894 cases, mean of 3,780 cases). As a consequence, one plausible explanation for many of these results is “small sample size”—especially any result that highlights the absence of a correlation.

There are larger GWAS summary statistics available for many of these cancers, see e.g <https://github.com/xueyaowunci/cancer-context-fine-mapping>. Results from analyses on larger GWAS will be more robust.

Response: We thank the reviewer for the comment. For the cancers listed in the GitHub folder, four (ER+ breast cancer, ER- breast cancer, endometrial cancer, and esophageal cancer) were not included in this study. For breast, lung, and prostate cancer, the sample sizes of GWASs used in our study were larger. For colorectal, renal, and bladder cancer, the GWASs listed in GitHub contained the UK Biobank and thus were not eligible for our study.

The ovarian cancer GWAS had a substantially larger sample size than the one used in our original analysis, so we incorporated it into the revised study. The updated GWAS yielded a higher SNP heritability estimate (0.09, P = 3.36 × 10^-6^) compared with the previous GWAS (0.006, P = 3.68 × 10^-3^), indicating improved statistical power. In the genome-wide genetic correlation analysis, ovarian cancer showed a significant genetic correlation with breast cancer, but not with bladder cancer (Figure 2), consistent with previous studies. Ovarian cancer showed local genetic correlation with several other cancers, while no mutually correlated regions containing ovarian cancer were found (Supplementary Table 3). In the PRS analysis, the updated ovarian cancer PRS was significantly associated with ovarian cancer itself (Supplementary Table 9), but no additional cross-cancer PRS associations were observed. The manuscript has been revised accordingly.

Supplementary Table 1. GWAS summary statistics.

Figure 2. Genome-wide genetic correlations across cancers.

Supplementary Table 3. Significant local genetic correlations (Part).

Supplementary Table 9. Associations between PRS and target cancers.

We have also incorporated the limited power into the limitations in lines 560-562: “Of note, the relatively modest sample sizes of several source GWASs may have limited statistical power to detect shared genetic signals, particularly for less common cancers.”

Specific comments follow

1. Methods:

1.1 The details in the methods section are a little thin, which makes it difficult for the reader to assess or interpret the results without reading cited methodological or data resources. The authors don’t need to recapitulate all of the information in the original papers, but some important high-level details would be very helpful.

For example:

–Provide URLs and date accessed and/or persistent identifier for cancer GWAS summary statistics;

Response: We have added a column named ‘Source Study’ in Supplementary Table 1 to indicate an identifier for cancer GWAS summary statistics.

ST1. GWAS Summary Statistics

–Provide an overview of how the FinnGen PanCancer GWAS was run (any record of any cancer ever versus no record of any cancer ever?)

Response: We have added a description of the pan-cancer GWAS in line 67: “The pan-cancer was defined as the diagnosis of any malignant neoplasm.”

–Clarify the definition of controls in the UK Biobank. “...participants without a recorded diagnosis of cancer”--does that mean diagnosis of *any* cancer, or of the target cancer?

Response: We are sorry for the confusion and have clarified in lines 87-89: “Individuals with any cancer diagnosis were classified as cases, and controls were defined as participants without a recorded diagnosis of any cancers.”

–In FinnGen and UKB, are cases incident after blood draw or a mix of prevalent and incident cases?

Response: Cases included diagnoses before and after enrollment in FinnGen and UKB. For common variant analysis, especially large-scale genome-wide studies, it was widely recognized that the common inherited risk was stable across the lifetime. Therefore, we believed that the inclusion of prevalent cases should have minimal effects on our results. To clarify, we added this as a limitation in lines 546-550: “Furthermore, the cancer GWASs and UKB analyses included prevalent cancer cases diagnosed before genotyping. Because germline genetic risk is fixed at birth, this design is unlikely to substantially affect the estimated inherited susceptibility. Nevertheless, future studies restricted to incident cancer cases would be valuable to further evaluate the robustness of our findings.”

–Does SDPR generate “infinitessimal” PRSes (i.e. every variant has a non-zero weight although some may be extremely shrunk) or does it learn a sparsity parameter (i.e. some percentage of variants get zero weight)?

Response: We thank the reviewer for this question. SDPR applies a Dirichlet process prior on the variance of SNP effect sizes and behaves much more like an infinitesimal model, where effect sizes of some SNPs can be heavily shrunk toward zero but not necessarily exactly zero.

We have added the description in lines 134-136: “SDPR employs a Dirichlet process prior on SNP effect-size variances, resulting in continuous shrinkage of effect estimates toward zero without explicitly assuming a fixed proportion of causal variants.”

1.2. On FinnGen, other than sample size:

–Use of FinnGen’s “PanCancer” results may complicate interpretation: if the outcome in the PanCacer GWAS is diagnosis with any cancer, then of course cancers A, B, and C will be genetically correlated with the PanCancer trait, even if the genetic contributions to A, B, and C are orthogonal.

Response: We agree that the pan-cancer phenotype includes cases from individual cancer types and therefore positive genetic correlations between a specific cancer and pan-cancer may partially reflect phenotype overlap. Accordingly, we do not interpret these associations as direct evidence of shared biological mechanisms among cancer types. Instead, we emphasize the observed genetic correlations as evidence of shared genetic susceptibility between one cancer and overall cancer risk. While some degree of positive correlation is expected because individual cancers contribute to the pan-cancer phenotype, the magnitude of these correlations remains informative and is not predetermined.

–I wonder whether the correlations with cancers whose summary stats do not come from FinnGen are deflated due to mis-match in genetic variation between sampled populations. Minor allele frequencies and linkage disequilibrium patterns will differ between large aggregate European ancestry samples and Finnish samples. This is always an issue when comparing summary statistics from different studies, and the further the genetic distance of the compared studies, the bigger the potential problem. FinnGen-EUR is not as large a mismatch as EAS-EUR, e.g., but might be enough to matter (esp combined with small sample size). Bringing in other large European ancestry GWAS summary statistics might help here, but the potential confounding of genetic correlations by sample ancestry differences should be mentioned as a limitation.

Response: We thank the reviewer for the suggestion and have added it to the limitations in lines 550-553: “Additionally, differences in genetic ancestry across the included GWASs, particularly between FinnGen and other European-ancestry studies, may have influenced the estimated genetic correlations. Future studies using more ancestrally homogeneous datasets will be valuable for validating and refining these estimates.”

1.3. On the use of “supercontrols:”

Using “supercontrols” (individuals without a personal history of *any* cancer) in the FinnGen individual cancer analyses (and perhaps also in the UK Biobank analyses) will, in principle, induce associations between the target cancer and variants related to cancers other than the target cancer. (Folks never diagnosed with cancers B, C, and D likely carry fewer risk alleles for cancers B, C, and D.) This could induce genetic correlations among cancers, especially more common cancers (ahem, NMSC). The magnitude of this potential bias is unclear, but the possibility should be noted.

Response: We agree that the use of cancer-free controls may theoretically induce modest positive correlations among cancer-specific GWASs because controls are depleted of alleles associated with other cancer types. However, such effects are expected to be limited because variants associated exclusively with one cancer are unlikely to show strong associations with unrelated cancers.

Nevertheless, we acknowledge that this design may contribute to some inflation of cross-cancer genetic correlations and have added this to the limitations in lines 553-557: “Sixth, the use of cancer-free controls in several source GWASs may have modestly influenced estimates of cross-cancer genetic correlation because controls were depleted of risk alleles associated with other cancer types. Future studies using alternative control definitions may help further evaluate the impact of this design choice on pleiotropy estimates.”

1.4. When assessing the association between the PRS for cancer A and cancer B, the authors adjust for the PRS for cancer B. The authors give no justification for this adjustment, and I’m not sure it is appropriate in this context. For variants that are truly associated with both cancers, would this adjustment (inappropriately) weaken the correlation between the PRS for A and cancer B? For variants that are only associated with cancer A but happen to be linkage disequilibrium (LD) with a variant truly associated with B, would this procedure actually remove the confounding due to LD? Those are honest Qs. Please provide some motivation for this adjustment and justification that it does what it should be doing.

Response: We thank the reviewer for the questions and apologize for the confusion. The motivation of adjusting PRS for target cancer is to determine whether the PRS for cancer B was associated with cancer A beyond the genetic liability already captured by the PRS for cancer A. We agree that conditioning on the target cancer PRS may attenuate associations arising from shared genetic variants between the two cancers. However, this was intentional, as we sought to identify cross-cancer genetic associations that provide predictive information independent of the target cancer PRS. We have clarified this motivation in lines 147-148: “The inclusion of PRS for cancer *i* was intended to evaluate whether the PRS for cancer *j* provided information on the risk of cancer *i* beyond that explained by the PRS for cancer *i*.”

1.5. Similarly, I didn’t understand the justification for removing individuals with multiple cancers. The authors note that “associations could be driven by comorbidity between cancers,” but, as I read that, “comorbidity” is a synonym for pleiotropy and hence a feature, not a bug. It‘s part of the phenomenon motivating this whole exercise. Maybe the authors are trying to rule out the situation where G -> cancer A -> cancer B, but (a) that’s still of interest, (b) I suspect that is not a widespread phenomenon, (c) if that’s the rationale, the order of diagnoses matters and that is not considered here, and (d) many folks with multiple cancers got them because of some underlying dysfunction that is independently raising risk for both cancers–excluding those people is excluding the people who are very informative for understanding pleiotropy. Not only are the genetic correlations estimated after removing the overlap noisier, they are systematically attenuated towards the null–which I think is an artifact of removing individuals with multiple cancers.

Response: We appreciate the reviewer’s comment and agree that individuals with multiple cancer diagnoses may be enriched for pleiotropic mechanisms. However, the primary objective of this study was to identify shared polygenic susceptibility across cancers rather than associations driven by cancer comorbidity itself. Multiple cancer diagnoses may arise through mechanisms beyond common polygenic risk, including high-penetrance rare germline variants, somatic alterations, treatment-related effects, or shared environmental exposures. Therefore, we performed a sensitivity analysis excluding individuals with multiple cancers and prioritized findings that remained significant after exclusion. While this approach may attenuate some true pleiotropic signals, it increases confidence that the reported associations reflect shared common polygenic susceptibility rather than being predominantly driven by individuals with multiple cancer diagnoses.

We have further clarified the rationale in lines 151-155: “Because cancer comorbidity may be influenced by factors beyond shared common polygenic susceptibility, including rare variants, somatic alterations, and environmental exposures, we conducted sensitivity analyses excluding individuals diagnosed with both cancers to assess the robustness of the observed associations.”

We have also added this to the limitations in lines 557-559: “Additionally, the exclusion of individuals with multiple cancer diagnoses in sensitivity analyses may have reduced the ability to detect certain pleiotropic effects, as these individuals could be enriched for shared genetic susceptibility across cancers.”

1.6. How were pleiotropy groups defined? How is genetic correlation with a pleiotropy group estimated? I’m interested in reading the preprint (ref. 24), but until I do I have to guess at what was done here.

Response: We thank the reviewer for the questions. These pleiotropy groups were defined using domain knowledge of phenotype relationships, with their biological relevance further supported by significant genetic correlations among grouped phenotypes. We have added the explanation in lines 169-171: “These groups were defined using domain knowledge of phenotype relationships, with their biological relevance further supported by significant genetic correlations among grouped phenotypes”.

1.7. Comparing Nagelkerke’s R2 between Equations 1, 2 and 3:

–How was missing data (if any) handled? If different numbers of individuals were included in fitting models 1-3, comparing Nagelkerke’s R2s will be invalid.

Response: We apologize for the lack of clarity. The same set of individuals was used for all three models, and no missing data were present for the variables included in these analyses. Therefore, the comparisons of Nagelkerke’s R^2^ across models were based on identical study samples and are valid.

–Was the permutation test done so as to preserve the correlation between cancer i (but not j) and age, sex, and top 10 PCs? If so, how? If not, this procedure is invalid, because the observed data includes any real-world associations between cancer i and the covariates but the permuted data does not.

Response: We thank the reviewer for these insightful questions. In the original analysis, the permutation procedure did not preserve the associations between cancer *i* and the covariates. Following the reviewer’s suggestion, we revised the permutation procedure to maintain these relationships under the null model. Specifically, we first fit the null model including the PRS for cancer *i* and all covariates, estimated the corresponding outcome probabilities, and then generated permuted outcomes by resampling from these fitted probabilities while preserving the overall number of cases. This approach retains the associations of the outcome with the PRS for cancer *i* and covariates under the null hypothesis while breaking the association with the tested PRS. The revised analysis yielded results highly consistent with the original findings (Figure 4). The manuscript has been revised accordingly in lines 192-199: “To test the null hypothesis that the across-cancer association cannot be attributed to the PD-PRS, a permutation test was conducted with 1,000 permutations while preserving the associations between the outcome, the PRS for cancer *i*, and covariates. Specifically, we first fitted the null model including the PRS for cancer *i* and all covariates and estimated the corresponding outcome probabilities. Permuted outcomes were then generated by resampling from these fitted probabilities while preserving the overall number of cases. The association between the PD-PRS and the permuted outcomes was subsequently evaluated to obtain the empirical null distribution”.

Figure 4. Attenuation of explained variance (∆R^2^) for PD-PRSs.

1.8. “Unless otherwise specified, multiple testing was controlled using Bonferroni correction.” Just cut this, as the Bonferroni correction used varies from analysis to analysis (as the number of tests varies from analysis to analysis). You’ve done a good job saying what the thresholds used were for various analyses and why, so this blanket statement is unnecessary..

Response: We thank the reviewer for the suggestion and have revised the manuscript accordingly by removing the sentence.

2. Results:

2.1. Seems to be a formatting error at the bottom of page 12. Some text has gone mising?

Response: We apologize for any confusion. We carefully reviewed the manuscript and did not identify any missing text or formatting issues at the bottom of page 12 in the version available to us. It is possible that the issue arose during the file conversion or review process.

2.2. Page 13. “Two cancer pairs with significant genome-wide correlations did not show any significant local genetic contributions, which suggests the shared genetic signal for these pairs is likely distributed across the genome rather than driven by specific regions.” Does it? It also seems likely that this could be due to limited power. These two pairs have the smallest number of cases among the pairs with significant genome-wide correlations. Absence of evidence is not evidence of absence.

Response: We thank the reviewer and agree that the limited power of GWASs could be an alternative explanation. We have clarified it in lines 240-245: “In contrast, the MSC-NHL pair, which was significantly genetically correlated, did not show any significant local genetic correlations. This discrepancy may reflect limited power to detect local genetic correlations for these cancers, a polygenic shared genetic architecture distributed across many genomic regions, or a combination of both factors”. In addition, we have added the limited power of GWASs as a limitation in lines 559-561: “Of note, the relatively modest sample sizes of several source GWASs may have limited statistical power to detect shared genetic signals, particularly for less common cancers.”

2.3. Page 14 and Table 1. Including a “landmark” (gene or cytoband) in Table 1 will help many readers who haven’t memorized chr:pos for all regions of interest, esp known multi-cancer loci (e.g. 1q25, 2q33/CASP8, HLA, 8q24/MYC, 6q27, 9p22, 11q12). The cancers for 2q33 are repeated; typo?

Response: We thank the reviewer for the suggestion and have added the cytoband column to Table 1. For 6p21 and 11q12, two mutually correlated clusters were identified. For a better visualization, we added borderlines to each region.

Table 1. Selected regions with mutually correlated cancer clusters.

Why exclude the HLA-overlapping region from downstream analysis?

Response: We thank the reviewer for this comment. The HLA region was excluded because of its exceptionally complex LD structure and high degree of polymorphism, which can lead to unstable estimates and complicate the interpretation of local genetic correlation analyses. Exclusion of the HLA region is a common practice in genome-wide and local genetic correlation studies for this reason, especially in LDSC, which requires accurate estimates of LD scores. Our objective was to identify robust shared polygenic signals across the genome while minimizing the potential influence of a single region with unusually complex genetic architecture.

We have clarified this rationale in lines 254-258: “and one region on chromosome 6 (chr6:32424108-32682443; 6p21) was excluded due to overlap with the major histocompatibility complex (MHC) since its extensive and complex LD structure can lead to unstable estimates and complicate the interpretation of local genetic correlation analyses”.

Worth commenting here or in discussion on what’s known already about these loci: e.g. Lindstrom JNCI found individual variants at 2q33 pleiotropic for breast, prostate, and melanoma; at HLA with lung, prostate, head and neck, colorectal, endometrial, and lung cancers; at 8q24 with breast, prostate, and colorectal; and at 11q12 with breast and prostate cancer; Kar Cancer Discovery 2016 found evidence for pleiotropy with breast, prostate and ovarian at 11q12; Kong (<https://www.biorxiv.org/content/10.64898/2026.01.06.697866v1> )reviews evidence for associations at 2q33 with breast cancer, melanoma, NMSC,and lung cancer; etc.

Response: We thank the reviewer for the helpful references. We note that region 2q33 did not meet the predefined eligibility criteria and was therefore excluded from subsequent analyses. For the regions of interest that were retained, we have expanded the Results section to discuss existing evidence supporting their involvement in cancer susceptibility, as follows:

For 6q27 in lines 274-276: “This region has previously been associated with basal cell carcinoma [26], and may represent a locus with broader relevance to cancer risk.”

For 8q24 in lines 282-284: “The clustering of breast, colorectal, and prostate cancers at 8q24 is consistent with previous reports [8], further supporting the role of this region as a shared cancer susceptibility locus.”

For 9p22 in lines 289-291: “This region is an established risk region for ovarian cancer [27], and our results further extended it beyond a single cancer.”

For 11q12 in lines 296-298: “This region has previously been associated with breast, prostate, and ovarian cancer risk [8,28]. Our findings further supported its role as a shared cancer susceptibility locus.”

For 1q25 in lines 302-304: “Similar associations have been established for colorectal and prostate cancers previously [31,32], whereas our findings suggest a broader role for this locus in shared cancer susceptibility.”

More striking is the absence of some well-documented multi-cancer loci, such as TERT and ABO. Why were these not identified? Is this a result of the statistical method, the input data, or both?

Response: We thank the reviewer for these questions. We examined the regions containing TERT and ABO in our local genetic correlation results. For TERT, the gene was split across two adjacent predefined regions (chr5:10,056-1,267,356 and chr5:1,270,983-1,762,678 in hg19), rather than being fully captured within a single region. This regional definition may have reduced the power to detect local genetic correlation at the TERT locus.

For the ABO gene, there was one block (chr9: 135401721-136417159) that covered the gene range, but no strong local genetic correlations were found. In Lindstrom et al. (2023), they found this gene to be pleiotropic for breast, ovarian, and pancreatic cancers. The failure to identify it in our analyses might be related to the limited power of the GWASs used for ovarian and pancreatic cancers.

We have added the discussion of limitations in lines 533-537: “Second, some established pleiotropic cancer loci may not have been detected because of limitations of the predefined regional partitioning, as illustrated by the *TERT* gene being split across two adjacent regions (chr5:10,056-1,267,356 and chr5:1,270,983-1,762,678), or because of limited power and heterogeneity in the input GWAS summary statistics, as observed for the *ABO* gene.”

2.4. Page 14. For genetic correlations with GWAS results from the Neale lab, please cite the Phenotype Code. For GWAS catalog look ups, please cite the EFO ID.

Response: We thank the reviewer for the suggestion. Accordingly, we have added phenotype code to Supplementary Tables 4-7 and EFO information to Supplementary Table 8.

ST4. Correlations with non-cancer phenotypes in chr6:167178790-168548525

ST5. Correlations with non-cancer phenotypes in chr8:128166556-128542444

ST6. Correlations with non-cancer phenotypes in chr9:18660695-19129349

ST7. Correlations with non-cancer phenotypes in chr11:63154309-66835194

ST8. GWAS Catalog associations for selected regions

2.5. Page 14. “Variants were enriched for four cancer-related GWAS traits.” These are not cancer-related traits, they are cancers. You can say “Variants were enriched for four cancers” and list them.

Response: We thank the reviewer for the correction and apologize for the confusion. We have revised the sentence in lines 280-282: “Variants were enriched for four cancers including urinary bladder carcinoma, breast carcinoma, CLL, and prostate carcinoma (Supplementary Table 8).”

2.6. Page 15. “three cancers were correlated,” “four cancers were correlated”: list them.

Response: We appreciate the suggestion and have added cancers to lines 286-287: “For chr9:18660695-19129349 (9p22), three cancers (lung cancer, prostate cancer, and thyroid gland cancer) were significantly correlated with 18 non-cancer phenotypes” and lines 293-294: “In the chr11:63154309–66835194 (11q12) region, four cancers (colorectal cancer, breast cancer, NHL, and NMSC) were correlated with 30 non-cancer phenotypes”

2.7. Page 16. “To further evaluate potential confounding by smoking…” (a) Is this confounding or mediation? If the genetic contributions to bladder cancer and lung cancer are distinct and are not correlated with smoking, then we should not see a genetic correlation between the cancers. If the genetic components are correlated with smoking, then there would be a genetic correlation–via the mechanism of smoking (which is still of interest). (b) Failure to find “significant” associations between lung cancer and the bladder cancer PRS may just be a power thing; the effect estimates are similar across smoking strata and in the pooled analysis. I don’t know that removing the lung-bladder PRS pair from downstream analysis is warranted; nor do I think you can conclude based on p-values (one of which just missed “significance”) that these results “indicate context specific patterns of polygenic association.”

Response: We thank the reviewer for the comments. (a) We agree that the term ‘confounding’ was imprecise in this context and that smoking is a potential mediator or shared pathway through which genetic susceptibility may influence the risk of both cancers. The objective of this analysis was rather to assess whether the observed cross-cancer PRS associations were primarily driven by smoking-related mechanisms or remained evident among individuals with similar smoking exposure. We have clarified the rationale in the manuscript accordingly in lines 333-334: “To further evaluate whether the identified shared polygenic risk can be fully attributed to smoking”.

(b) We agree that loss of statistical significance in stratified analyses may reflect reduced power rather than true heterogeneity, particularly when effect estimates are similar across strata. We therefore revised our interpretation and no longer describe these findings as evidence of context-specific polygenic associations. In addition, we retained the bladder cancer PRS-lung cancer pair in downstream analyses, while noting that smoking-related mechanisms may contribute to this association. We have revised the manuscript accordingly in lines 337-341: “In contrast, associations between lung cancer and the bladder cancer PRS were no longer significant in either stratum, but the effect estimates were similar to those observed in the pooled analysis (Supplementary Table 10), indicating potential loss of power in stratified analyses. Our results suggest that smoking-related mechanisms alone are unlikely to account for the observed genetic overlap between bladder and lung cancers.”.

We have added PD-PRS and PS-PRS results for the lung cancer-bladder cancer PRS pair in the following lines:

Lines 364-365: “For bladder cancer, associations with alcohol consumption- and hypertension-related PD-PRSs failed to reach significance (Supplementary Figure 3A).”

Lines 371-373: “For bladder cancer, the neuropsychiatric disease PD-PRS showed the strongest association (OR = 1.29, 95% CI: 1.26-1.33), implicating enrichment of related function in the development of bladder cancer.”

Lines 393-395: “While lung cancer-bladder cancer PRS pair, the diabetes PD-PRS for bladder cancer was not associated with lung cancer risk (OR = 1.02, 95% CI: 0.99-1.04), and the lipids PD-PRS showed the strongest association (OR = 1.04, 95% CI: 1.01-1.07).”

Lines 413-416: “For bladder cancer risk, lung cancer PD-PRSs related to atherosclerotic disease, chronic kidney disease, diabetes, and neuropsychiatric disease showed significantly greater $\Delta R^{2}$, while for lung cancer, all five associated bladder cancer PD-PRSs related to atherosclerotic disease, autoimmune disease, chronic kidney disease, lipids, and neuropsychiatric disease showed significantly greater $\Delta R^{2}.$”

Lines 449-451: “Similar pathways were found for associations between bladder cancer PS-PRS and lung cancer risk (Supplementary Figure 4E-F), further supporting the roles of these pathways in genetic overlap for these two cancers.”

2.8. Page 17. It took me several reads and flipping back and forth between pages to understand why you only focused on the lung, NMSC, and CLL PRS. For thick people like me, you might make this explicit. “We selected the PRS involved in these cancer-PRS pairs (lung, NMSC, and CLL PRS) for downstream analysis. We applied two decomposition frameworks to these PRS to further characterize…”

Response: We thank the reviewer for the comment and apologize for the confusion. The manuscript has been revised accordingly in lines 348-351: “PRSs involved in the six selected cancer pairs—lung cancer PRS, bladder cancer PRS, NMSC PRS, and CLL PRS—were involved for the following decompositions. Specifically, we applied two decomposition frameworks to the four selected genome-wide PRSs to further characterize sources of shared polygenic risk.”

2.9. Page 18. “Consistent with metabolic contributions to lung cancer.” Reference? Are metabolic changes (e.g. metabolic syndrome) causally related to lung cancer (which is how I read “contributes to”) or are they associated with lung cancer (markers of latent disease development)?

Response: We apologize for the confusion. We have clarified and added the reference in lines 376-378: “For lung cancer, the diabetes PD-PRS exhibited the largest effect size (OR = 1.27, 95% CI: 1.23-1.30), consistent with evidence linking metabolic dysfunction, insulin resistance, and diabetes-related pathways to cancer development and progression [30]”.

2.10. Page 19. “To formally assess…” I have concerns about the validity of these analyses (see 1.7 above). And even if they were valid, I am not sure what they add beyond the PD-PRS results presented in the previous paragraph.

Response: We thank the reviewer for the comment. The permutation analysis has been revised to account for outcome-covariate association structures as explained above.

The purpose of this analysis was not to identify additional associations, but to **quantify the relative contribution** of individual PD-PRS components to observed cross-cancer PRS associations. While association analyses identify pleiotropy groups linked to cancer risk, they do not indicate whether those groups account for a substantial proportion of the cross-cancer signal. The attenuation analysis addresses this question by quantifying the loss of predictive information when a specific PD-PRS component is removed from the source cancer PRS. We have clarified this rationale in lines 419-421: “Overall, decomposing genome-wide cancer PRSs into pleiotropy-informed components enabled quantification of relative contributions of specific phenotype groups to cross-cancer associations.”.

3. Discussion, page 23: “the shared polygenic signal between bladder and lung cancers is enriched… for inflammatory traits, such as cardiovascular and neuropsychiatric phenotypes, which have been implicated in cancer susceptibility.” (a) Neuropsychiatric traits are inflammatory? Maybe, in part, as these are complex traits that involve multiple systems. But using that standard there are few complex diseases that are not inflammatory. More importantly, because the definition of neuropsychiatric traits is not given here (it’s in a reference preprint), it’s hard to evaluate this statement. (b) Language is unclear. “...which have been implicated…”: what does “which” refer to here? Inflammatory traits? Or cardiovascular and neuropsychiatric traits? (c) Are these the best references here? They are not specific to bladder or lung cancer, and focus on shared CVD & cancer risk factors (not CVD and cancer per se) or on neuroinflammation specifically (as opposed to neuropsychiatric traits broadly)? There’s also a bit of conflation of cancer (treatment) sequelae and cancer risk factors in those refs.

Response: We thank the reviewer for these helpful comments. (a) We agree that inflammation is not the most appropriate mechanism to emphasize based on the current analyses and have revised the discussion accordingly. Rather than supporting a specific inflammatory pathway, our results consistently indicate that the shared polygenic susceptibility between bladder and lung cancers is enriched within interconnected functional domains identified through both PD-PRS and PS-PRS analyses. Thus, while the mechanistic interpretation has been refined, the central conclusion—that shared genetic risk is concentrated within specific biological functions rather than distributed uniformly across the genome—remains supported by multiple complementary analyses.

(b) The ‘which’ here refers to the cardiovascular and neuropsychiatric diseases, and we have clarified this in the manuscript.

(c) We agree that the cited references were not essential to support our main interpretation. The key finding was that the shared polygenic susceptibility between bladder and lung cancers was enriched within cardiovascular and neuropsychiatric phenotype groups identified through the PD-PRS framework. To avoid overinterpretation, we removed the references and revised the discussion to focus on the results directly supported by our analyses.

To clarify, we have revised the manuscript in lines 513-522: “Specifically, our PD-PRS analyses indicate that the shared polygenic signal between bladder and lung cancers is enriched for genetic components associated with cardiovascular and neuropsychiatric phenotypes. Complementary PS-PRS analyses similarly highlight enrichment in immune- and virus-related pathways. Although these findings do not implicate a single causal mechanism, they suggest that the shared genetic susceptibility between bladder and lung cancers may arise from interconnected biological processes involving multiple functions. These findings provide a structured framework for interpreting cross-cancer PRS associations and suggest that shared polygenic risk is concentrated within specific functional domains rather than distributed uniformly across the genome.”

Reviewer #2: This manuscript investigates the shared genetic risk across cancers. Through genetic correlation and polygenic risk score analyses, the paper demonstrated that although genome-wide sharing is modest, there are specific regions with stronger sharing of cross-cancer polygenic risk. Overall, the paper addresses the interesting question and uses rigorous methods. The manuscript is generally well written. However, some of the findings are hard to interpret. I have the following comments.

Response: We thank the reviewer for the comments and hope our responses addressed the concerns.

1. Some text at the bottom of page 12, GWAS summary statistics, seems to be missing due to document formatting issues. Please add back the text.

Response: We apologize for any confusion. We carefully reviewed the manuscript and did not identify any missing text or formatting issues at the bottom of page 12 in the version available to us. It is possible that the issue arose during the file conversion or review process.

2. Page 14, paragraphs 2 and 3: The authors mentioned local genetic correlation between cancers total protein levels. However, the interpretation is unclear. Is there previous literature that discusses the link between cancer and total protein levels? What are plausible hypotheses?

Response: We thank the reviewer for the questions. To clarify the results, we added the non-cancer phenotype code in Supplementary Table 4. In addition, we have improved the interpretation by linking our findings to previous evidence in lines 268-272: “Although total protein itself is not an established cancer risk factor, it reflects the combined abundance of circulating proteins and may capture underlying immune and inflammatory processes. This interpretation is supported by previous prospective proteomic studies demonstrating associations between numerous plasma proteins and the future risk of lung cancer and NHL [27].”

3. Page 15, paragraph 3: Variants chr1:182294372–183796074 were mapped to chronotype-related phenotypes. The authors should comment on the connection between chronotype and cancer.

Response: We have revised the manuscript accordingly to improve interpretations in lines 302-304: “Similar associations have been established for colorectal and prostate cancers previously [31,32], whereas our findings suggest a broader role for this locus in shared cancer susceptibility.”

4. Page 18, paragraph 1: “For NMSC, the neuropsychiatric disease PD-PRS showed the
strongest association (OR = 1.46, 95% CI: 1.44–1.48), suggesting enrichment of shared genetic effects between NMSC and neuropsychiatric phenotypes at the polygenic level.” This finding seems difficulty to interpret – what are the hypotheses explaining shared genetic architecture between NMSC and neuropsychiatric disorders?

Response: We thank the reviewer and apologize for the confusion. We have added more interpretations and also improved the clarity in lines 379-386: “For NMSC, the neuropsychiatric disease PD-PRS showed the strongest association (OR = 1.46, 95% CI: 1.44–1.48), suggesting that genetic components enriched for neuropsychiatric disease may also contribute to NMSC susceptibility. Although the underlying mechanisms remain unclear, previous studies have reported shared genetic architecture between neurological disorders such as Parkinson’s disease and skin cancers, potentially reflecting overlap in pigmentation biology, neural crest development, DNA damage response, and immune regulation [34].”

5. The acronyms of some cancers, such as CLL, NHL, are not defined in the main text. Although they are defined in the supplements, it would improve readability to define them in the main text where they first appear.

Response: We thank the reviewer for the suggestion and have revised in the manuscript accordingly.

6. The font size in Figures 3 and 5 is very small. Consider increasing it to improve legibility.

Response: We have increased the font size in Figures 3 and 5.

Figure 3. Cross-cancer associations of PD-PRS.

Figure 5. Significant cross-cancer associations of PS-PRS.
