# Supplementary material for: Identifying shared polygenic risk across cancers
Source: PLoS Genet. 2026 Sep 18;22(9):e1012308. doi: 10.1371/journal.pgen.1012308 (PMC13600620; doi:10.1371/journal.pgen.1012308)
Supplement: Peer review history

# Peer review: Decision Letter 0

**DOI:** 10.1371/journal.pgen.1012308.r001

**Authors:** Kent W. Hunter, Heather J Cordell

12 May 2026

PGENETICS-D-26-00272

Identifying shared polygenic risk across cancers

PLOS Genetics

Dear Dr. Zhao,

Thank you for submitting your manuscript to PLOS Genetics. After careful consideration, we feel that it has merit but does not fully meet PLOS Genetics's publication criteria as it currently stands. Therefore, we invite you to submit a revised version of the manuscript that addresses the points raised during the review process.

Please submit your revised manuscript by Jul 11 2026 11:59PM. If you will need more time than this to complete your revisions, please reply to this message or contact the journal office at plosgenetics@plos.org. When you're ready to submit your revision, log on to https://www.editorialmanager.com/pgenetics/ and select the 'Submissions Needing Revision' folder to locate your manuscript file.

Please include the following items when submitting your revised manuscript:

* A letter that responds to each point raised by the editor and reviewer(s). You should upload this letter as a separate file labeled 'Response to Reviewers'. This file does not need to include responses to any formatting updates and technical items listed in the 'Journal Requirements' section below.

* A marked-up copy of your manuscript that highlights changes made to the original version. You should upload this as a separate file labeled 'Revised Manuscript with Track Changes'.

* An unmarked version of your revised paper without tracked changes. You should upload this as a separate file labeled 'Manuscript'.

If you would like to make changes to your financial disclosure, competing interests statement, or data availability statement, please make these updates within the submission form at the time of resubmission. Guidelines for resubmitting your figure files are available below the reviewer comments at the end of this letter.

As the corresponding author, your ORCID iD is verified in the submission system and will appear in the published article. PLOS supports the use of ORCID, and we encourage all coauthors to register for an ORCID iD and use it as well. Please encourage your coauthors to verify their ORCID iD within the submission system before final acceptance, as unverified ORCID iDs will not appear in the published article. Only the individual author can complete the verification step; PLOS staff cannot verify ORCID iDs on behalf of authors.

We look forward to receiving your revised manuscript.

Kind regards,

Kent W. Hunter

Section Editor

PLOS Genetics

Kent Hunter

Section Editor

PLOS Genetics

Aimée Dudley

Editor-in-Chief

PLOS Genetics

Anne Goriely

Editor-in-Chief

PLOS Genetics

**Journal Requirements:**

1) Please ensure that the CRediT author contributions listed for every co-author are completed accurately and in full.

At this stage, the following Authors/Authors require contributions: jiaqi hu. Please ensure that the full contributions of each author are acknowledged in the "Add/Edit/Remove Authors" section of our submission form.

The list of CRediT author contributions may be found here: https://journals.plos.org/plosgenetics/s/authorship#loc-author-contributions

2) Please provide an Author Summary. This should appear in your manuscript between the Abstract (if applicable) and the Introduction, and should be 150-200 words long. The aim should be to make your findings accessible to a wide audience that includes both scientists and non-scientists. Sample summaries can be found on our website under Submission Guidelines:

https://journals.plos.org/plosgenetics/s/submission-guidelines#loc-parts-of-a-submission

3) Please upload all main figures as separate Figure files in .tif or .eps format. For more information about how to convert and format your figure files please see our guidelines:

https://journals.plos.org/plosgenetics/s/figures

4) We have noticed that you have uploaded Supporting Information files, but you have not included a list of legends. Please add a full list of legends for your Supporting Information files after the references list.

5) Thank you for stating that "The summary-level data (e.g. PRS weights) will be available on the PGS catalog (https://www.pgscatalog.org/) once published." We strongly recommend all authors deposit their data before acceptance, as the process can be lengthy and hold up publication timelines. Please note that, though access restrictions are acceptable now, your entire minimal dataset will need to be made freely accessible if your manuscript is accepted for publication. This policy applies to all data except where public deposition would breach compliance with the protocol approved by your research ethics board.

6) Your current Financial Disclosure states, "The author(s) received no specific funding for this work.".

However, your funding information on the submission form indicates receiving funds.Please ensure that the funders and grant numbers match between the Financial Disclosure field and the Funding Information tab in your submission form. Note that the funders must be provided in the same order in both places as well.

Please amend your detailed Financial Disclosure statement. This is published with the article. It must therefore be completed in full sentences and contain the exact wording you wish to be published.

1) Please clarify all sources of financial support for your study. List the grants, grant numbers, and organizations that funded your study, including funding received from your institution. Please note that suppliers of material support, including research materials, should be recognized in the Acknowledgements section rather than in the Financial Disclosure

2) State the initials, alongside each funding source, of each author to receive each grant. For example: "This work was supported by the National Institutes of Health (####### to AM; ###### to CJ) and the National Science Foundation (###### to AM)."

3) State what role the funders took in the study. If the funders had no role in your study, please state: "The funders had no role in study design, data collection and analysis, decision to publish, or preparation of the manuscript."

4) If any authors received a salary from any of your funders, please state which authors and which funders.

7) Please revise your current Competing Interest statement to the standard "The authors have declared that no competing interests exist."

Note: If the reviewer comments include a recommendation to cite specific previously published works, please review and evaluate these publications to determine whether they are relevant and should be cited. There is no requirement to cite these works unless the editor has indicated otherwise.

**Reviewers' comments:**

Reviewer's Responses to Questions

Reviewer #1: This paper uses publicly available summary genome-wide association data and individual-level data from the UK Biobank to assess the genetic correlations between cancers and decompose these correlations into putative functional components. The top-line findings are in line with previous studies [PMIDs 36929942 30683880 26464424 27432226]: although the overall genetic correlations between cancers are weak, there are regions with stronger correlations among multiple cancers (e.g. TERT, HLA, 8q24, ABO, others discussed below).The authors use these local correlations between cancers and correlations between cancers and other traits to assess the mechanisms driving these correlations. These analyses take an approach that has previously been used to understand the polygenic contribution to individual traits (including at least one cancer) [41315867 38374256 38443691 https://www.medrxiv.org/content/10.1101/2025.05.15.25327708v1] and apply it to multiple traits.

The paper is hard to follow in some places and could use more details in the methods and results sections (specifics below). Some of the authors’ interpretations of their results are “too little” (e.g. tautological restatements of the empirical results) and some are “too much,” latching on to one possible explanation for the results, without considering alternatives (again, specifics below). There are also some analytic choices that I would not have made, and I didn’t find the authors’ justification compelling: notably, the focus on analyses removing individuals with multiple cancers, which shifts the focus away from shared genetic contributions to distinct genetic and environmental contributions.

But the biggest limitation of this paper is the limited power in most of the input GWAS and the UK Biobank.The GWAS summary statistics for 12 of the 24 cancers they considered come from a single biobank, FinnGen, with small case numbers (median of 1,894 cases, mean of 3,780 cases). As a consequence, one plausible explanation for many of these results is “small sample size”—especially any result that highlights the absence of a correlation.

There are larger GWAS summary statistics available for many of these cancers, see e.g https://github.com/xueyaowunci/cancer-context-fine-mapping. Results from analyses on larger GWAS will be more robust.

Specific comments follow

1. Methods:

1.1 The details in the methods section are a little thin, which makes it difficult for the reader to assess or interpret the results without reading cited methodological or data resources. The authors don’t need to recapitulate all of the information in the original papers, but some important high-level details would be very helpful.

For example:

–Provide URLs and date accessed and/or persistent identifier for cancer GWAS summary statistics;

–Provide an overview of how the FinnGen PanCancer GWAS was run (any record of any cancer ever versus no record of any cancer ever?)

–Clarify the definition of controls in the UK Biobank. “...participants without a recorded diagnosis of cancer”--does that mean diagnosis of *any* cancer, or of the target cancer?

–In FinnGen and UKB, are cases incident after blood draw or a mix of prevalent and incident cases?

–Does SDPR generate “infinitessimal” PRSes (i.e. every variant has a non-zero weight although some may be extremely shrunk) or does it learn a sparsity parameter (i.e. some percentage of variants get zero weight)?

1.2. On FinnGen, other than sample size:

–Use of FinnGen’s “PanCancer” results may complicate interpretation: if the outcome in the PanCacer GWAS is diagnosis with any cancer, then of course cancers A, B, and C will be genetically correlated with the PanCancer trait, even if the genetic contributions to A, B, and C are orthogonal.

–I wonder whether the correlations with cancers whose summary stats do not come from FinnGen are deflated due to mis-match in genetic variation between sampled populations. Minor allele frequencies and linkage disequilibrium patterns will differ between large aggregate European ancestry samples and Finnish samples. This is always an issue when comparing summary statistics from different studies, and the further the genetic distance of the compared studies, the bigger the potential problem. FinnGen-EUR is not as large a mismatch as EAS-EUR, e.g., but might be enough to matter (esp combined with small sample size). Bringing in other large European ancestry GWAS summary statistics might help here, but the potential confounding of genetic correlations by sample ancestry differences should be mentioned as a limitation.

1.3. On the use of “supercontrols:”

Using “supercontrols” (individuals without a personal history of *any* cancer) in the FinnGen individual cancer analyses (and perhaps also in the UK Biobank analyses) will, in principle, induce associations between the target cancer and variants related to cancers other than the target cancer. (Folks never diagnosed with cancers B, C, and D likely carry fewer risk alleles for cancers B, C, and D.) This could induce genetic correlations among cancers, especially more common cancers (ahem, NMSC). The magnitude of this potential bias is unclear, but the possibility should be noted.

1.4. When assessing the association between the PRS for cancer A and cancer B, the authors adjust for the PRS for cancer B. The authors give no justification for this adjustment, and I’m not sure it is appropriate in this context. For variants that are truly associated with both cancers, would this adjustment (inappropriately) weaken the correlation between the PRS for A and cancer B? For variants that are only associated with cancer A but happen to be linkage disequilibrium (LD) with a variant truly associated with B, would this procedure actually remove the confounding due to LD? Those are honest Qs. Please provide some motivation for this adjustment and justification that it does what it should be doing.

1.5. Similarly, I didn’t understand the justification for removing individuals with multiple cancers. The authors note that “associations could be driven by comorbidity between cancers,” but, as I read that, “comorbidity” is a synonym for pleiotropy and hence a feature, not a bug. It‘s part of the phenomenon motivating this whole exercise. Maybe the authors are trying to rule out the situation where G -> cancer A -> cancer B, but (a) that’s still of interest, (b) I suspect that is not a widespread phenomenon, (c) if that’s the rationale, the order of diagnoses matters and that is not considered here, and (d) many folks with multiple cancers got them because of some underlying dysfunction that is independently raising risk for both cancers–excluding those people is excluding the people who are very informative for understanding pleiotropy. Not only are the genetic correlations estimated after removing the overlap noisier, they are systematically attenuated towards the null–which I think is an artifact of removing individuals with multiple cancers.

1.6. How were pleiotropy groups defined? How is genetic correlation with a pleiotropy group estimated? I’m interested in reading the preprint (ref. 24), but until I do I have to guess at what was done here.

1.7. Comparing Nagelkerke’s R2 between Equations 1, 2 and 3:

–How was missing data (if any) handled? If different numbers of individuals were included in fitting models 1-3, comparing Nagelkerke’s R2s will be invalid.

–Was the permutation test done so as to preserve the correlation between cancer i (but not j) and age, sex, and top 10 PCs? If so, how? If not, this procedure is invalid, because the observed data includes any real-world associations between cancer i and the covariates but the permuted data does not.

1.8. “Unless otherwise specified, multiple testing was controlled using Bonferroni correction.” Just cut this, as the Bonferroni correction used varies from analysis to analysis (as the number of tests varies from analysis to analysis). You’ve done a good job saying what the thresholds used were for various analyses and why, so this blanket statement is unnecessary..

2. Results:

2.1. Seems to be a formatting error at the bottom of page 12. Some text has gone mising?

2.2. Page 13. “Two cancer pairs with significant genome-wide correlations did not show any significant local genetic contributions, which suggests the shared genetic signal for these pairs is likely distributed across the genome rather than driven by specific regions.” Does it? It also seems likely that this could be due to limited power. These two pairs have the smallest number of cases among the pairs with significant genome-wide correlations. Absence of evidence is not evidence of absence.

2.3. Page 14 and Table 1. Including a “landmark” (gene or cytoband) in Table 1 will help many readers who haven’t memorized chr:pos for all regions of interest, esp known multi-cancer loci (e.g. 1q25, 2q33/CASP8, HLA, 8q24/MYC, 6q27, 9p22, 11q12). The cancers for 2q33 are repeated; typo?

Why exclude the HLA-overlapping region from downstream analysis?

Worth commenting here or in discussion on what’s known already about these loci: e.g. Lindstrom JNCI found individual variants at 2q33 pleiotropic for breast, prostate, and melanoma; at HLA with lung, prostate, head and neck, colorectal, endometrial, and lung cancers; at 8q24 with breast, prostate, and colorectal; and at 11q12 with breast and prostate cancer; Kar Cancer Discovery 2016 found evidence for pleiotropy with breast, prostate and ovarian at 11q12; Kong (https://www.biorxiv.org/content/10.64898/2026.01.06.697866v1 )reviews evidence for associations at 2q33 with breast cancer, melanoma, NMSC,and lung cancer; etc.

More striking is the absence of some well-documented multi-cancer loci, such as TERT and ABO. Why were these not identified? Is this a result of the statistical method, the input data, or both?

2.4. Page 14. For genetic correlations with GWAS results from the Neale lab, please cite the Phenotype Code. For GWAS catalog look ups, please cite the EFO ID.

2.5. Page 14. “Variants were enriched for four cancer-related GWAS traits.” These are not cancer-related traits, they are cancers. You can say “Variants were enriched for four cancers” and list them.

2.6. Page 15. “three cancers were correlated,” “four cancers were correlated”: list them.

2.7. Page 16. “To further evaluate potential confounding by smoking…” (a) Is this confounding or mediation? If the genetic contributions to bladder cancer and lung cancer are distinct and are not correlated with smoking, then we should not see a genetic correlation between the cancers. If the genetic components are correlated with smoking, then there would be a genetic correlation–via the mechanism of smoking (which is still of interest). (b) Failure to find “significant” associations between lung cancer and the bladder cancer PRS may just be a power thing; the effect estimates are similar across smoking strata and in the pooled analysis. I don’t know that removing the lung-bladder PRS pair from downstream analysis is warranted; nor do I think you can conclude based on p-values (one of which just missed “significance”) that these results “indicate context specific patterns of polygenic association.”

2.8. Page 17. It took me several reads and flipping back and forth between pages to understand why you only focused on the lung, NMSC and CLL PRS. For thick people like me, you might make this explicit. “We selected the PRS involved in these cancer-PRS pairs (lung, NMSC, and CLL PRS) for downstream analysis. We applied two decomposition frameworks to these PRS to further characterize…”

2.9. Page 18. “Consistent with metabolic contributions to lung cancer.” Reference? Are metabolic changes (e.g. metabolic syndrome) causally related to lung cancer (which is how I read “contributes to”) or are they associated with lung cancer (markers of latent disease development)?

2.10. Page 19. “To formally assess…” I have concerns about the validity of these analyses (see 1.7 above). And even if they were valid, I am not sure what they add beyond the PD-PRS results presented in the previous paragraph.

3. Discussion, page 23: “the shared polygenic signal between bladder and lung cancers is enriched… for inflammatory traits, such as cardiovascular and neuropsychiatric phenotypes, which have been implicated in cancer susceptibility.” (a) Neuropsychiatric traits are inflammatory? Maybe, in part, as these are complex traits that involve multiple systems. But using that standard there are few complex diseases that are not inflammatory. More importantly, because the definition of neuropsychiatric traits is not given here (it’s in a reference preprint), it’s hard to evaluate this statement. (b) Language is unclear. “...which have been implicated…”: what does “which” refer to here? Inflammatory traits? Or cardiovascular and neuropsychiatric traits? (c) Are these the best references here? They are not specific to bladder or lung cancer, and focus on shared CVD & cancer risk factors (not CVD and cancer per se) or on neuroinflammation specifically (as opposed to neuropsychiatric traits broadly)? There’s also a bit of conflation of cancer (treatment) sequelae and cancer risk factors in those refs.

Reviewer #2: This manuscript investigates the shared genetic risk across cancers. Through genetic correlation and polygenic risk score analyses, the paper demonstrated that although genome-wide sharing is modest, there are specific regions with stronger sharing of cross-cancer polygenic risk. Overall, the paper addresses the interesting question and uses rigorous methods. The manuscript is generally well written. However, some of the findings are hard to interpret. I have the following comments.

1. Some text at the bottom of page 12, GWAS summary statistics, seems to be missing due to document formatting issues. Please add back the text.

2. Page 14, paragraphs 2 and 3: The authors mentioned local genetic correlation between cancers total protein levels. However, the interpretation is unclear. Is there previous literature that discusses the link between cancer and total protein levels? What are plausible hypotheses?

3. Page 15, paragraph 3: Variants chr1:182294372–183796074 were mapped to chronotype-related phenotypes. The authors should comment on the connection between chronotype and cancer.

4. Page 18, paragraph 1: “For NMSC, the neuropsychiatric disease PD-PRS showed the

strongest association (OR = 1.46, 95% CI: 1.44–1.48), suggesting enrichment of shared genetic effects between NMSC and neuropsychiatric phenotypes at the polygenic level.” This finding seems difficulty to interpret – what are the hypotheses explaining shared genetic architecture between NMSC and neuropsychiatric disorders?

5. The acronyms of some cancers, such as CLL, NHL, are not defined in the main text. Although they are defined in the supplements, it would improve readability to define them in the main text where they first appear.

6. The font size in Figures 3 and 5 is very small. Consider increasing it to improve legibility.

**********

**Have all data underlying the figures and results presented in the manuscript been provided?**

Large-scale datasets should be made available via a public repository as described in the *PLOS Genetics*  data availability policy, and numerical data that underlies graphs or summary statistics should be provided in spreadsheet form as supporting information.

Reviewer #1: **No:**  The publicly available GWAS results are cited, but URLs and accession numbers are not given.

Reviewer #2: None

**********

PLOS authors have the option to publish the peer review history of their article (what does this mean?). If published, this will include your full peer review and any attached files.

If you choose “no”, your identity will remain anonymous but your review may still be made public.

**Do you want your identity to be public for this peer review?**  For information about this choice, including consent withdrawal, please see our Privacy Policy.

Reviewer #1: No

Reviewer #2: No

[NOTE: If reviewer comments were submitted as an attachment file, they will be attached to this email and accessible via the submission site. Please log into your account, locate the manuscript record, and check for the action link "View Attachments". If this link does not appear, there are no attachment files.]

**Figure resubmission:**

**Reproducibility:**

To enhance the reproducibility of your results, we recommend that authors of applicable studies deposit laboratory protocols in protocols.io, where a protocol can be assigned its own identifier (DOI) such that it can be cited independently in the future. Additionally, PLOS ONE offers an option to publish peer-reviewed clinical study protocols. Read more information on sharing protocols at https://plos.org/protocols?utm_medium=editorial-email&utm_source=authorletters&utm_campaign=protocols

# Author comment: Author response to Decision Letter 1

**DOI:** 10.1371/journal.pgen.1012308.r002

20 Jul 2026

Attachment Submitted filename: Response to review.docx

# Peer review: Decision Letter 1

**DOI:** 10.1371/journal.pgen.1012308.r003

**Authors:** Kent W. Hunter, Heather J Cordell

26 Aug 2026

Dear Dr Zhao,

We are pleased to inform you that your manuscript entitled "Identifying shared polygenic risk across cancers" has been editorially accepted for publication in PLOS Genetics. Congratulations!

Before your submission can be formally accepted and sent to production you will need to complete our formatting changes, which you will receive in a follow up email. Please be aware that it may take several days for you to receive this email; during this time no action is required by you. Please note: the accept date on your published article will reflect the date of this provisional acceptance, but your manuscript will not be scheduled for publication until the required changes have been made.

Once your paper is formally accepted, an uncorrected proof of your manuscript will be published online ahead of the final version, unless you’ve already opted out via the online submission form. If, for any reason, you do not want an earlier version of your manuscript published online or are unsure if you have already indicated as such, please let the journal staff know immediately at plosgenetics@plos.org.

In the meantime, please log into Editorial Manager at https://www.editorialmanager.com/pgenetics/, click the "Update My Information" link at the top of the page, and update your user information to ensure an efficient production and billing process. Note that PLOS requires an ORCID iD for all corresponding authors. Therefore, please ensure that you have an ORCID iD and that it is validated in Editorial Manager. To do this, go to ‘Update my Information’ (in the upper left-hand corner of the main menu), and click on the Fetch/Validate link next to the ORCID field.  This will take you to the ORCID site and allow you to create a new iD or authenticate a pre-existing iD in Editorial Manager.

If you have a press-related query, or would like to know about making your underlying data available (as you will be aware, this is required for publication), please see the end of this email. If your institution or institutions have a press office, please notify them about your upcoming article at this point, to enable them to help maximise its impact. Inform journal staff as soon as possible if you are preparing a press release for your article and need a publication date.

Thank you again for supporting open-access publishing; we are looking forward to publishing your work in PLOS Genetics!

Yours sincerely,

Heather J Cordell

Academic Editor

PLOS Genetics

Kent Hunter

Section Editor

PLOS Genetics

Aimée Dudley

Editor-in-Chief

PLOS Genetics

Anne Goriely

Editor-in-Chief

PLOS Genetics

www.plosgenetics.org

BlueSky: @plos.bsky.social

----------------------------------------------------

Comments from the reviewers (if applicable):

Reviewer's Responses to Questions

**Comments to the Authors:**

**Please note here if the review is uploaded as an attachment.**

Reviewer #2: The authors have addressed my comments.

**********

**Have all data underlying the figures and results presented in the manuscript been provided?**

Large-scale datasets should be made available via a public repository as described in the *PLOS Genetics*  data availability policy, and numerical data that underlies graphs or summary statistics should be provided in spreadsheet form as supporting information.

Reviewer #2: None

**********

PLOS authors have the option to publish the peer review history of their article (what does this mean?). If published, this will include your full peer review and any attached files.

If you choose “no”, your identity will remain anonymous but your review may still be made public.

**Do you want your identity to be public for this peer review?**  For information about this choice, including consent withdrawal, please see our Privacy Policy.

Reviewer #2: No

----------------------------------------------------

**Data Deposition**

If you have submitted a Research Article or Front Matter that has associated data that are not suitable for deposition in a subject-specific public repository (such as GenBank or ArrayExpress), one way to make that data available is to deposit it in the Dryad Digital Repository. As you may recall, we ask all authors to agree to make data available; this is one way to achieve that. A full list of recommended repositories can be found on our website.

The following link will take you to the Dryad record for your article, so you won't have to re‐enter its bibliographic information, and can upload your files directly:

http://datadryad.org/submit?journalID=pgenetics&manu=PGENETICS-D-26-00272R1

More information about depositing data in Dryad is available at http://www.datadryad.org/depositing. If you experience any difficulties in submitting your data, please contact help@datadryad.org for support.

Additionally, please be aware that our data availability policy requires that all numerical data underlying display items are included with the submission, and you will need to provide this before we can formally accept your manuscript, if not already present.

----------------------------------------------------

**Press Queries**

If you or your institution will be preparing press materials for this manuscript, or if you need to know your paper's publication date for media purposes, please inform the journal staff as soon as possible so that your submission can be scheduled accordingly. Your manuscript will remain under a strict press embargo until the publication date and time. This means an early version of your manuscript will not be published ahead of your final version. PLOS Genetics may also choose to issue a press release for your article. If there's anything the journal should know or you'd like more information, please get in touch via plosgenetics@plos.org.

# Editor report: Acceptance letter

**DOI:** 10.1371/journal.pgen.1012308.r004

**Authors:** Kent W. Hunter, Heather J Cordell

PGENETICS-D-26-00272R1

Identifying shared polygenic risk across cancers

Dear Dr Zhao,

We are pleased to inform you that your manuscript entitled "Identifying shared polygenic risk across cancers" has been formally accepted for publication in PLOS Genetics! Your manuscript is now with our production department and you will be notified of the publication date in due course.

The corresponding author will soon be receiving a typeset proof for review, to ensure errors have not been introduced during production. Please review the PDF proof of your manuscript carefully, as this is the last chance to correct any errors. Please note that major changes, or those which affect the scientific understanding of the work, will likely cause delays to the publication date of your manuscript.

Soon after your final files are uploaded, unless you have opted out or your manuscript is a front-matter piece, the early version of your manuscript will be published online. The date of the early version will be your article's publication date. The final article will be published to the same URL, and all versions of the paper will be accessible to readers.

For Research Articles, you will receive an invoice from PLOS for your publication fee after your manuscript has reached the completed accept phase. If you receive an email requesting payment before acceptance or for any other service, this may be a phishing scheme. Learn how to identify phishing emails and protect your accounts at https://explore.plos.org/phishing.

Thank you again for supporting PLOS Genetics and open-access publishing. We are looking forward to publishing your work!

With kind regards,

Janani Seenivasan

PLOS Genetics

On behalf of:

The PLOS Genetics Team

Carlyle House, Carlyle Road, Cambridge CB4 3DN | United Kingdom

plosgenetics@plos.org | +44 (0) 1223-442823

plosgenetics.org | Twitter: @PLOSGenetics
